# Supplementary material for: MosAIC: An annotated collection of mosquito-associated bacteria with high-quality genome assemblies
Source: PLoS Biol. 2024 Nov 15;22(11):e3002897. doi: 10.1371/journal.pbio.3002897 (PMC11633956; doi:10.1371/journal.pbio.3002897)
Supplement: S2 Table — Metadata category names and definitions follow those presented in S1 Table. NA = not applicable. (DOCX) [file pbio.3002897.s002.docx]

| **Table S2.** Summary table of mosquito-bacterial origin. Metadata category names and definitions follow those presented in Table S1. NA = not applicable. | | | | | | | | | | | | | | | | |
| --- | --- | --- | --- | --- | --- | --- | --- | --- | --- | --- | --- | --- | --- | --- | --- | --- |
| **Mosquito species** |  | **Lab/field derived** | |  | **Life stage** | | |  | **Tissue**  (larvae/adults only) | |  | **Sex**  (adults only) | |  | **Feeding status**  (females only) | |
|  |  | **lab** | **field** |  | **eggs** | **larvae** | **adults** |  | **midgut** | **whole body** |  | **male** | **female** |  | **blood fed** | **non blood fed** |
| *Aedes aegypti* |  | 105 | 7 |  | 7 | 12 | 93 |  | 39 | 66 |  | 0 | 93 |  | 45 | 48 |
| *Aedes albopictus* |  | 11 | 60 |  | 0 | 6 | 65 |  | 9 | 62 |  | 26 | 39 |  | 0 | 39 |
| *Aedes atropalpus* |  | 5 | 0 |  | 0 | 5 | 0 |  | 0 | 5 |  | NA | NA |  | NA | NA |
| *Aedes taeniorhynchus* |  | 0 | 5 |  | 0 | 0 | 5 |  | 0 | 5 |  | 0 | 5 |  | 0 | 5 |
| *Aedes triseriatus* |  | 8 | 4 |  | 0 | 8 | 4 |  | 0 | 12 |  | 0 | 4 |  | 0 | 4 |
| *Aedes trivittatus* |  | 0 | 1 |  | 0 | 0 | 1 |  | 1 | 0 |  | 0 | 1 |  | 0 | 1 |
| *Anopheles crucians* |  | 0 | 3 |  | 0 | 0 | 3 |  | 0 | 3 |  | 0 | 3 |  | 0 | 3 |
| *Anopheles gambiae* |  | 15 | 0 |  | 0 | 0 | 15 |  | 11 | 4 |  | 0 | 15 |  | 6 | 9 |
| *Anopheles punctipennis* |  | 0 | 1 |  | 0 | 0 | 1 |  | 1 | 0 |  | 0 | 1 |  | 0 | 1 |
| *Anopheles quadrimaculatus* |  | 0 | 19 |  | 0 | 0 | 19 |  | 19 | 0 |  | 0 | 19 |  | 0 | 19 |
| *Anopheles* sp. |  | 0 | 2 |  | 0 | 0 | 2 |  | 0 | 2 |  | 0 | 2 |  | 0 | 2 |
| *Culex erraticus* |  | 0 | 1 |  | 0 | 0 | 1 |  | 1 | 0 |  | 0 | 1 |  | 0 | 1 |
| *Culex nigripalpus* |  | 0 | 9 |  | 0 | 0 | 9 |  | 0 | 9 |  | 0 | 9 |  | 0 | 9 |
| *Culex pipiens* |  | 15 | 8 |  | 0 | 6 | 17 |  | 9 | 14 |  | 0 | 17 |  | 0 | 17 |
| *Deinocerites cancer* |  | 0 | 4 |  | 0 | 0 | 4 |  | 0 | 4 |  | 0 | 4 |  | 0 | 4 |
| *Toxorhynchites amboinensis* |  | 10 | 0 |  | 0 | 6 | 4 |  | 0 | 10 |  | 0 | 4 |  | 0 | 4 |
| Unknown |  | 0 | 2 |  | 0 | 0 | 2 |  | 0 | 2 |  | 0 | 2 |  | 0 | 2 |
|  |  |  |  |  |  |  |  |  |  |  |  |  |  |  |  |  |
| ***Total*** |  | ***169*** | ***126*** |  | ***7*** | ***43*** | ***245*** |  | ***90*** | ***198*** |  | ***26*** | ***219*** |  | ***51*** | ***168*** |
