# Supplementary material for: MosAIC: An annotated collection of mosquito-associated bacteria with high-quality genome assemblies
Source: PLoS Biol. 2024 Nov 15;22(11):e3002897. doi: 10.1371/journal.pbio.3002897 (PMC11633956; doi:10.1371/journal.pbio.3002897)
Supplement: S1 File — (DOCX) [file pbio.3002897.s007.docx]

Supplementary File 1: Consortium Author List

2022 UW-Madison Capstone in Microbiology Students

Consortium authors include all persons enrolled in MICROBIO 551 (Capstone Research Project in Microbiology, 2 cr.) during the Spring 2022 semester that (*i*) were identified by Timothy Paustian (Teaching Professor, Department of Bacteriology) or Michelle Rondon (Teaching Faculty, Department of Bacteriology) as deserving co-authorship credit, and (*ii*) gave consent to have their name published alongside this study. These individuals are listed alphabetically below.

| Shraddha R. Bhide |  |  |
| --- | --- | --- |
| Annika H. Borgaonkar |  |  |
| Jakob J. Burkett |  |  |
| Daniel J. Chacko |  |  |
| Nikolas L. Christoffel |  |  |
| Joy Chung |  |  |
| Andrew J. DeMarco |  |  |
| Nicholas P. Durst |  |  |
| Pratyusha Emkay |  |  |
| Rebecca N. Forman |  |  |
| Adalee K. Gill |  |  |
| Uma A. Gude |  |  |
| Giovanni M. Hanstad |  |  |
| Lars H. Johnston |  |  |
| Matthew R. Johnston |  |  |
| Arman Kamrani |  |  |
| Martin Kelty |  |  |
| Noah R. Kokko-Ludemann |  |  |
| Kennah E. Konrad |  |  |
| Michael D. LeClaire |  |  |
| Grace Lin |  |  |
| Robert C. Londono |  |  |
| Jillian M. Lucito |  |  |
| Katelyn R. Major |  |  |
| Thomas J. Montana |  |  |
| John G. Price |  |  |
| Sarah A. Sabinash |  |  |
| Ann M. Saucedo |  |  |
| Allison A. Schopf |  |  |
| Anna Schwenn |  |  |
| Natalee H. Skoubis |  |  |
| Dawson J. Slusser |  |  |
| Justin Stensloff |  |  |
| Dianne L. Tebbe |  |  |
| Addison Vang |  |  |
