# Supplementary material for: MosAIC: An annotated collection of mosquito-associated bacteria with high-quality genome assemblies
Source: PLoS Biol. 2024 Nov 15;22(11):e3002897. doi: 10.1371/journal.pbio.3002897 (PMC11633956; doi:10.1371/journal.pbio.3002897)
Supplement: S2 File — (DOCX) [file pbio.3002897.s008.docx]

**Overview**

MosAIC (Mosquito-Associated Isolate Collection) is a resource consisting of 392 bacterial isolates from mosquitoes. These isolates come with extensive metadata and high-quality draft genome assemblies, publicly available for use by the scientific community. Please see https://kcoonlab.bact.wisc.edu/mosaic/ for more information.

**Analysis Overview**

Scripts for each analysis are written in R. Each directory contains the necessary files and code to recreate each figure of the manuscript. To repeat the analysis, clone the repository, and run each script. Do not `cd` into the cloned repository.

**Example**

Create a new project in RStudio. To run the scripts in 01_GenomeQC to recreate Figure 1B in the manuscript.

- Navigate to the terminal window and `git clone https://github.com/MosAIC-Collection/MosAIC_V1`.
- Open the script `checkM_analysis.R` from the files panel window.
- Install required packages.
- `cmd enter` from line `1`.

**Recreate the Manuscript Figures**

Once the repository has been cloned (above), recreate each figure opening and running each script as follows:

- ***Fig 1 Origin of bacterial isolates in MosAIC***
  - Fig 1 - Script: `04_Sankey_Diagram/Metadata_Snakey_Diagram.R`: run code from line `1` to `127`
- ***Fig 2 Phylogeny of single species representatives from MosAIC, along with quality-assurance metrics for related genome assemblies***
  - Fig 2A - Script: `03_MosAIC_Phylogeny/plot_tree_metadata.R`: run code from line `1` to `257`
  - Fig 2B - Script: `01_Genome_QC/checkM_analysis.R`: run code from line `1 to `60`
  - Fig 2C - Script: `01_Genome_QC/checkM_analysis.R`: run code from line `1` to `239`
  - Fig 2D - Script: `01_Genome_QC/checkM_analysis.R`: run code from line `1` to `136`
  - Fig 2E - Script: `01_Genome_QC/checkM_analysis.R`: run code from line `1` to `159`
  - Fig 2F - Script: `02_GTDB_Drep_Summary/gtdbtk_drep_stat.R`: run code from line `1` to `112`
- ***Fig 3 Heatmap of the distribution of virulence factors across all MosAIC genomes***
  - Fig 3 - Script: `05_Virulence_Factor_Analysis.R`: run code from line `1` to `192`
- ***Fig 4 Selected genus population structures with improved mosquito representation***
  - Fig 4A-C - Script: `06b_EnterobacterPopulationStructure/Enterobacter_Pop_Struc.R`: run code from line `1` to `426`
  - Fig 4D-F - Script: `06a_SerratiaPopulationStructure/Serratia_Genus_Pop_Structure.R`: run code from line `1` to `370`
  - Fig 4G-H - Script: `06c_ElizabethkingiaPopulationStructure/ElizabethkingiaPopStruc.R`: run code from line `1` to `300`
- ***Fig 5 Pangenomes of Enterobacter asburiae, Serratia marcescens, and Elizabethkingia anophelis with highlighted mosquito-associated lineages***
  - Fig 5A - Script: `07b_EnterobacterPangenome/EnterobacterPangenomeTree.R`: run code from line `1` to `249`
  - Fig 5B - Script: `07a_SerratiaPangenome/SerratiaMPangenome.R`: run code from line `1` to `210`
  - Fig 5C - Script: `07c_ElizabethkingiaPangenome/Elizabethkingia_Anophelis_Pangenome.R`: run code from line `1` to `177`

**Supplementary Figures**

- Fig S1 - Script: `01_Genome_QC/plot_QUAST.R`: run code from line `1` to `55`
- Fig S2 - Script: `01_Genome_QC/checkM_analysis.R`: run code from line `1` to `184`
- Fig S3 - Script: `02_GTDB_Drep_Summary.R`: run code from line `1` to `188`
- Fig S4 - Script: `12_Metadata_Exploration.R`: run code from line `1` to `148`
- Fig S5 - Script: `12_Metadata_Exploration.R`: run code from line `1` to `255`
- Fig S6 - Script: `05_Virulence_Factor_Analysis.R`: run code from line `1` to `340`
- Fig S7 - Script: `06b_EnterobacterPopulationStructure.R`: run code from line `1` to `154`
- Fig S8 - Script: `06a_SerratiaPopulationStructure.R`: run code from line `1` to `103`
- Fig S9 - Script: `06c_ElizabethkingiaPopulationStructure.R`: run code from line `1` to `105`
- Fig S10 - Script: `08_GeneAccumulationCurves.R`: run code from line `1` to `42`
- Fig S11-13 - Script: `10_VisPopPUNKClusters.R`: run code from line `1` to `67`
- Fig S14 - Script: `11_LineageCoreGeneAnalysis.R`: run code from line `1` to `167`
- Fig S15 - Script: `11_LineageCoreGeneAnalysis.R`: run code from line `1` to `293`

**Authors**

Aidan Foo - aidanfoo96@gmail.com

Laura Brettell - L.E.Brettell1@salford.ac.uk

Eva Heinz - eva.heinz@strath.ac.uk

Kerri Coon - kerri.coon@wisc.edu

**Citation**

Foo A, Brettell LE, Nichols HL, 2022 UW-Madison Capstone in Microbiology Students, Medina Muñoz M, Lysne JA, et al. Establishment and comparative genomics of a high-quality collection of mosquito-associated bacterial isolates - MosAIC (Mosquito-Associated Isolate Collection). 2023 Oct. Available from: http://biorxiv.org/lookup/doi/10.1101/2023.10.04.560816
