# Supplementary figures and images for: MosAIC: An annotated collection of mosquito-associated bacteria with high-quality genome assemblies
Source: PLoS Biol. 2024 Nov 15;22(11):e3002897. doi: 10.1371/journal.pbio.3002897 (PMC11633956; doi:10.1371/journal.pbio.3002897)

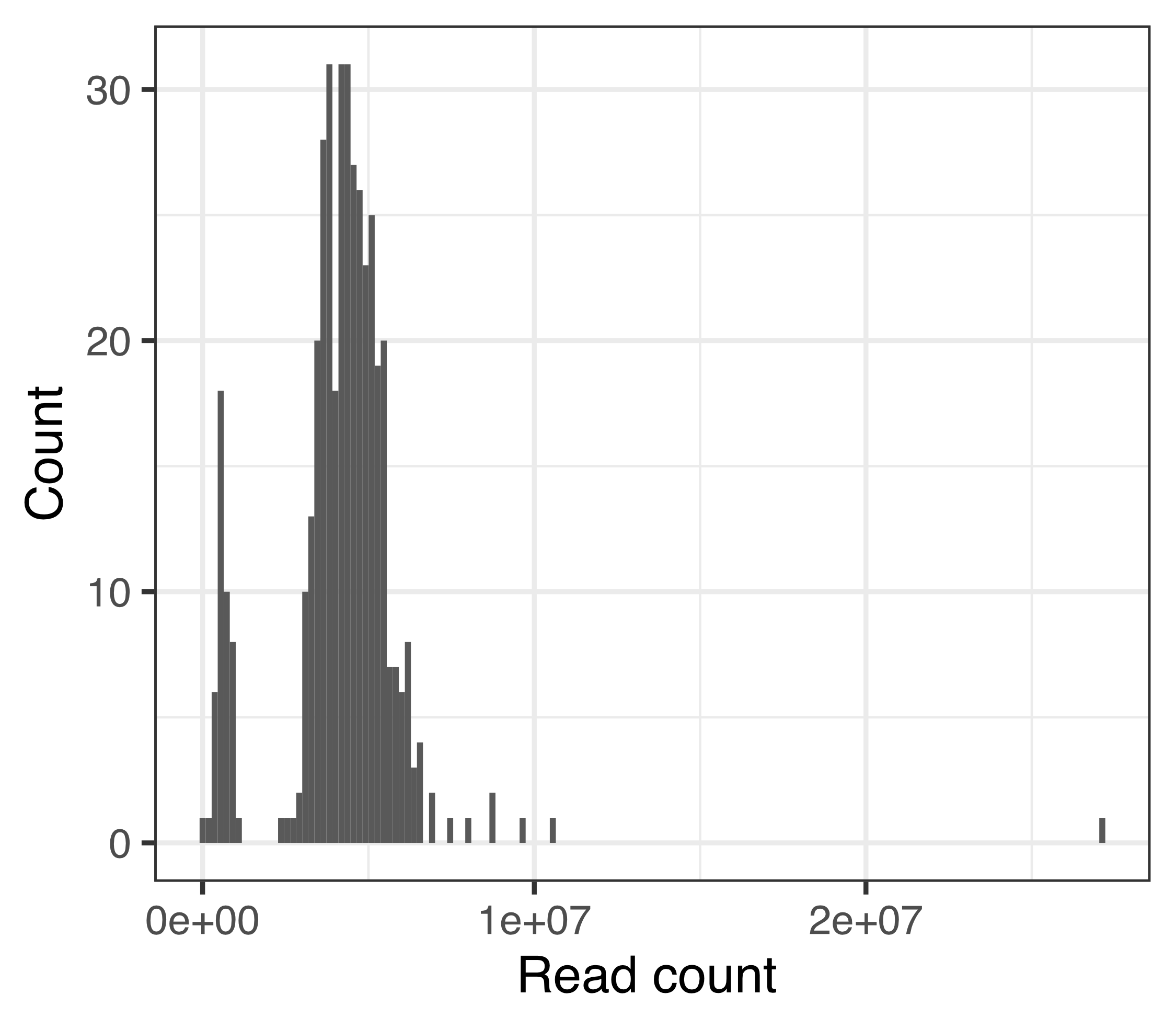

Supplement: S1 Fig — Histogram showing the size distribution of raw sequencing reads used to assemble MosAIC genomes. The x-axis shows the number of reads per isolate, while the y-axis shows the number of isolates with a specific read count. All code and data to recreate this figure can be found at https://github.com/MosAIC-Collection/MosAIC_V1 in folder “01_GenomeQC.” (TIFF) [file pbio.3002897.s009.tiff]

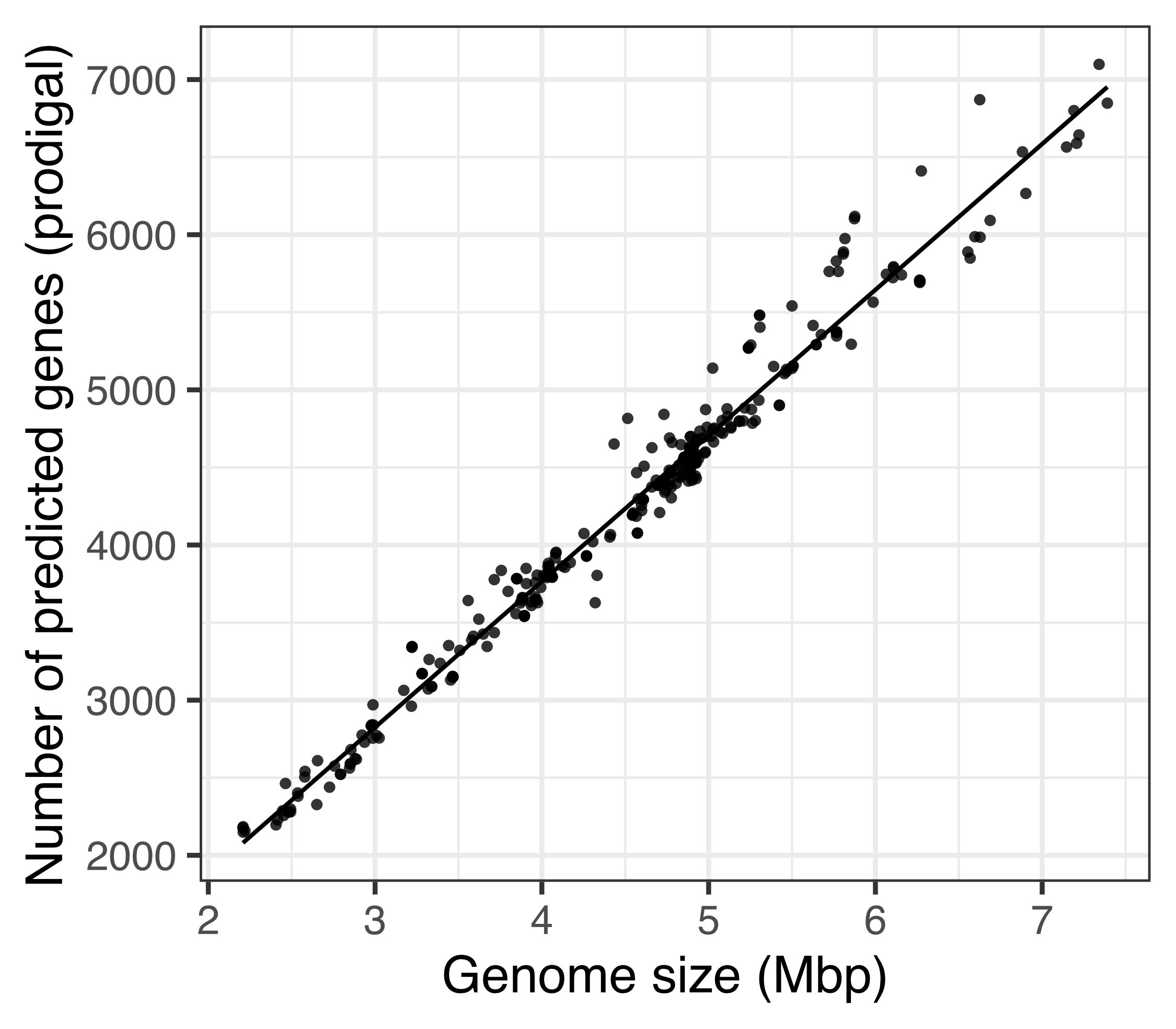

Supplement: S2 Fig — Scatterplot showing the relationship between genome size (x-axis) and number of predicted genes (y-axis) for each isolate. Each point represents a high-quality genome assembly (>CheckM completeness 98%, 10× read coverage). Line fitted using a linear model in R. Mbp = megabase pairs. All code and data to recreate this figure can be found at https://github.com/MosAIC-Collection/MosAIC_V1 in folder “01_GenomeQC.” (TIFF) [file pbio.3002897.s010.tiff]

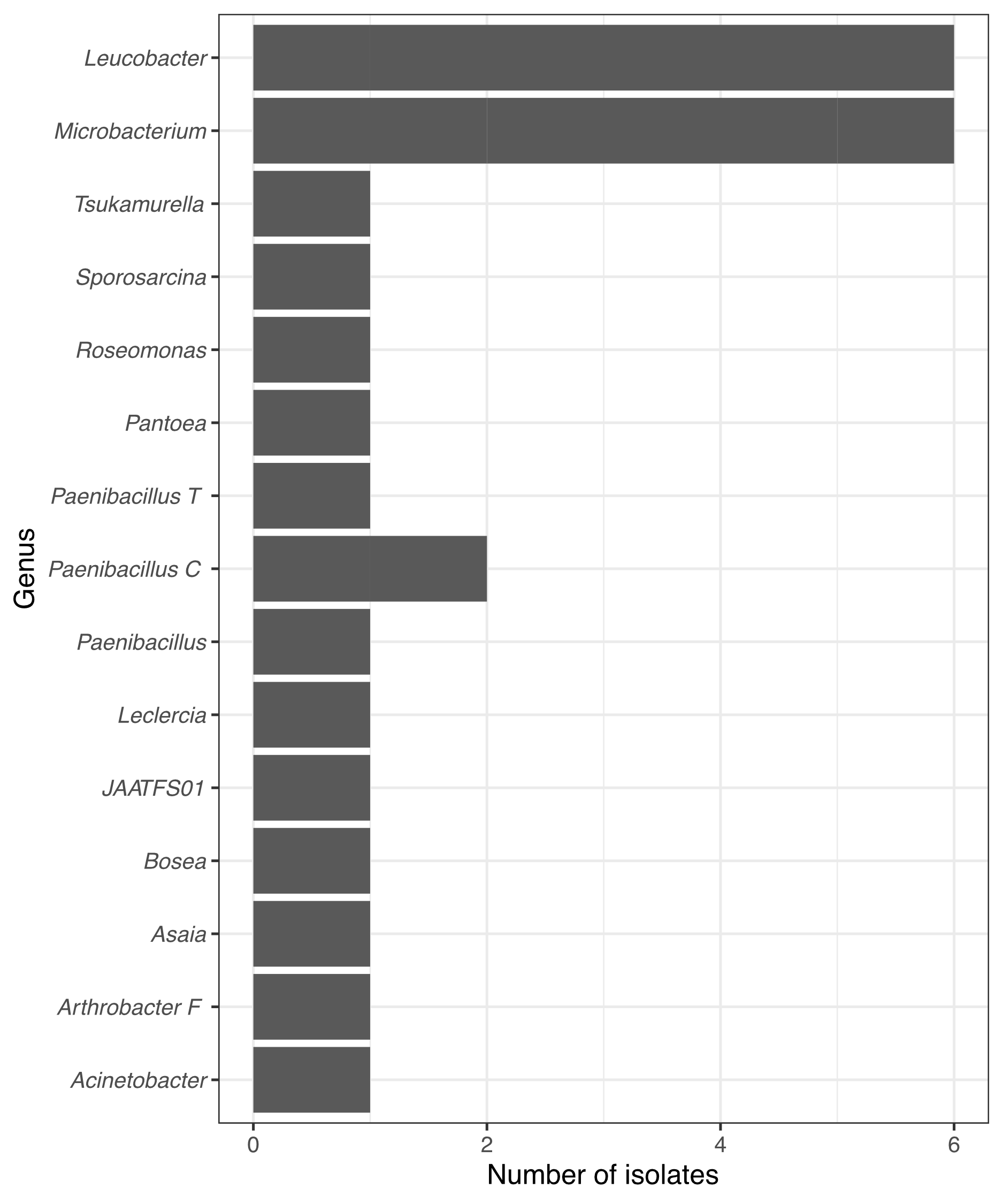

Supplement: S3 Fig — The x-axis of the bar chart shows the number of isolates assigned to a reference genome with a given genus assigned taxonomy in the GTDB (y-axis). “JAATFS01” is a strain identifier placeholder used by GTDB-Tk when no binomially named representative genome is present in the GTDB. All code and data to recreate this figure can be found at https://github.com/MosAIC-Collection/MosAIC_V1 in folder “02_GTDB_Drep_Summary.” (TIFF) [file pbio.3002897.s011.tiff]

A

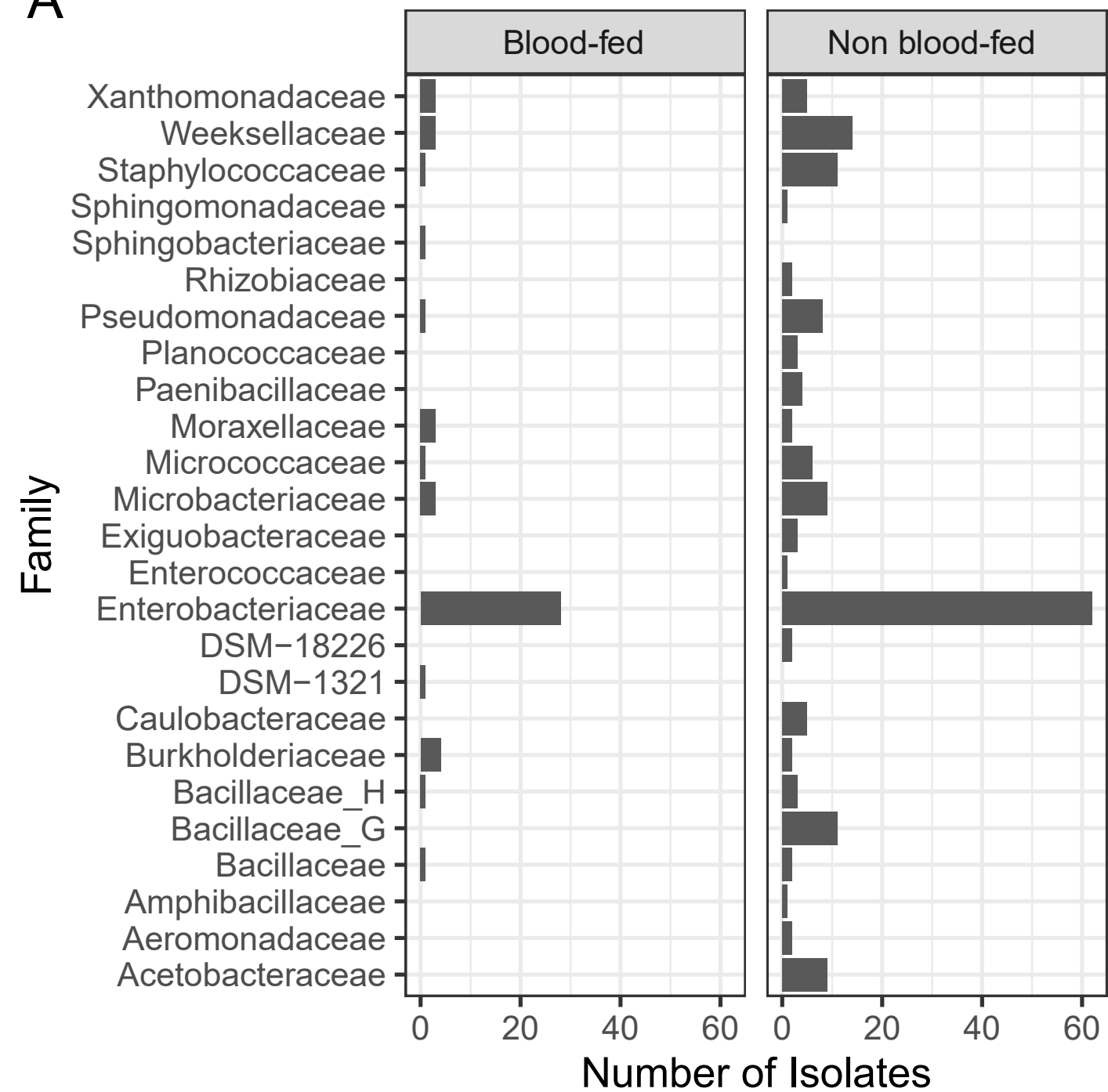

B

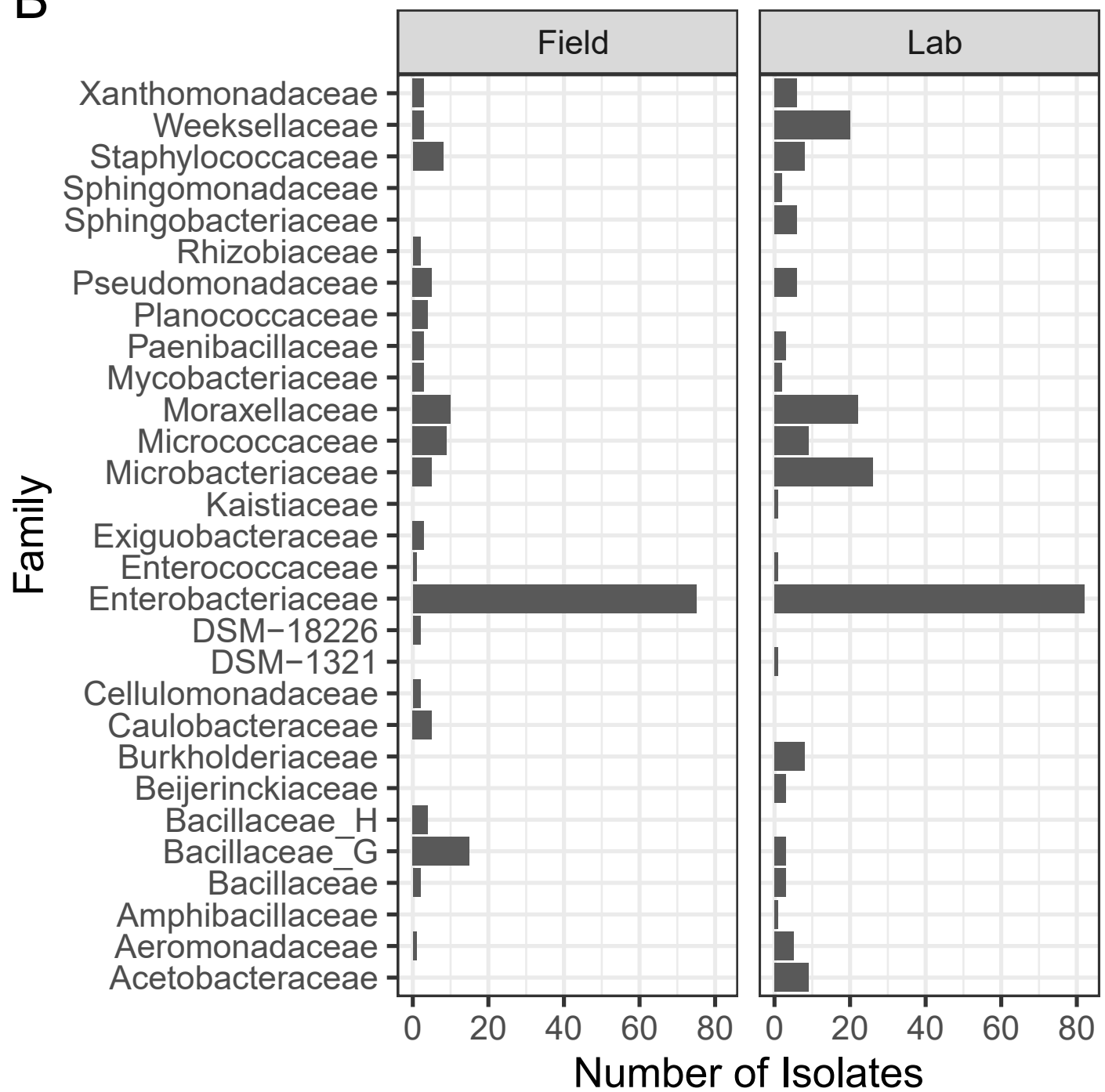

C

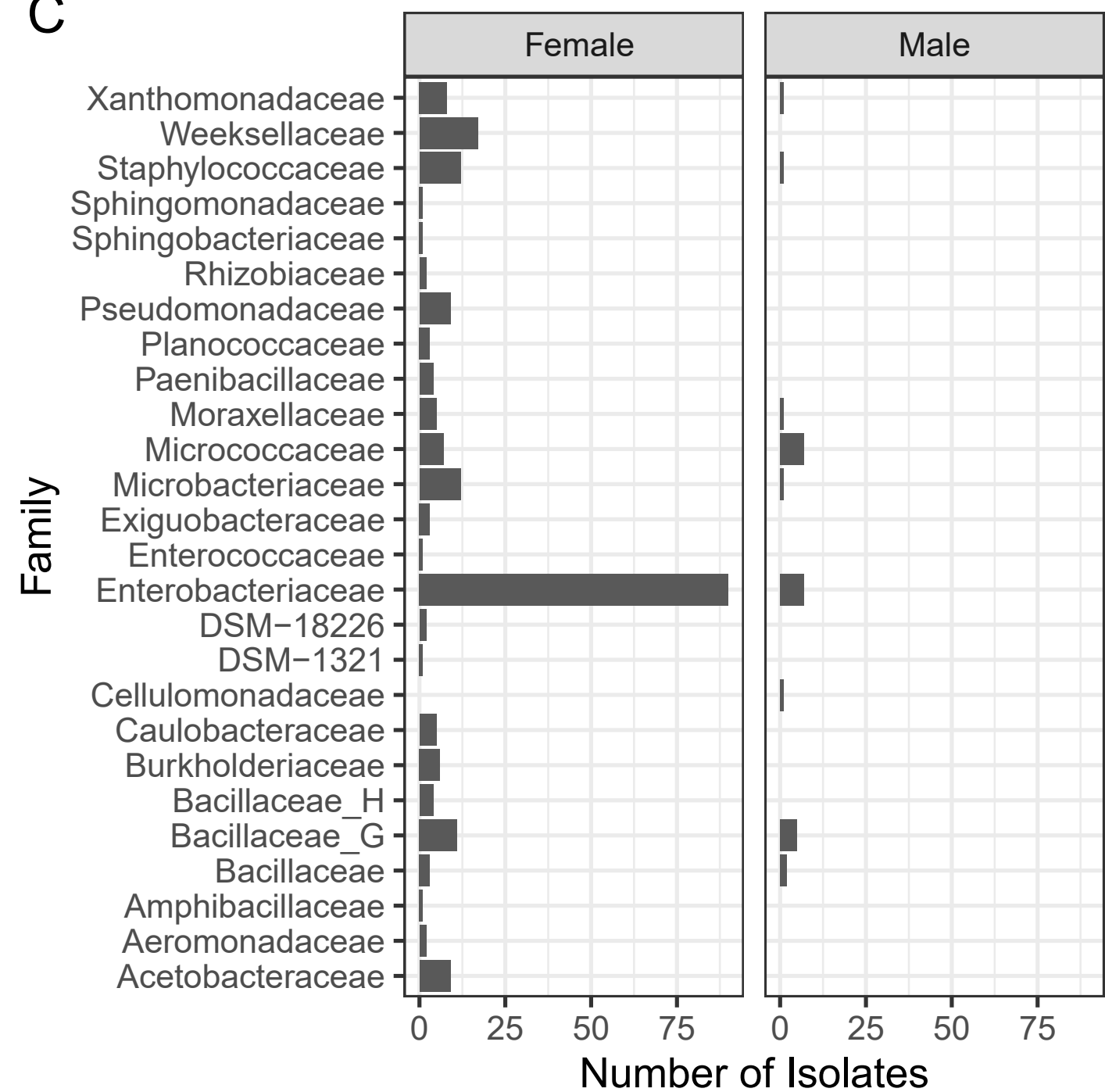

D

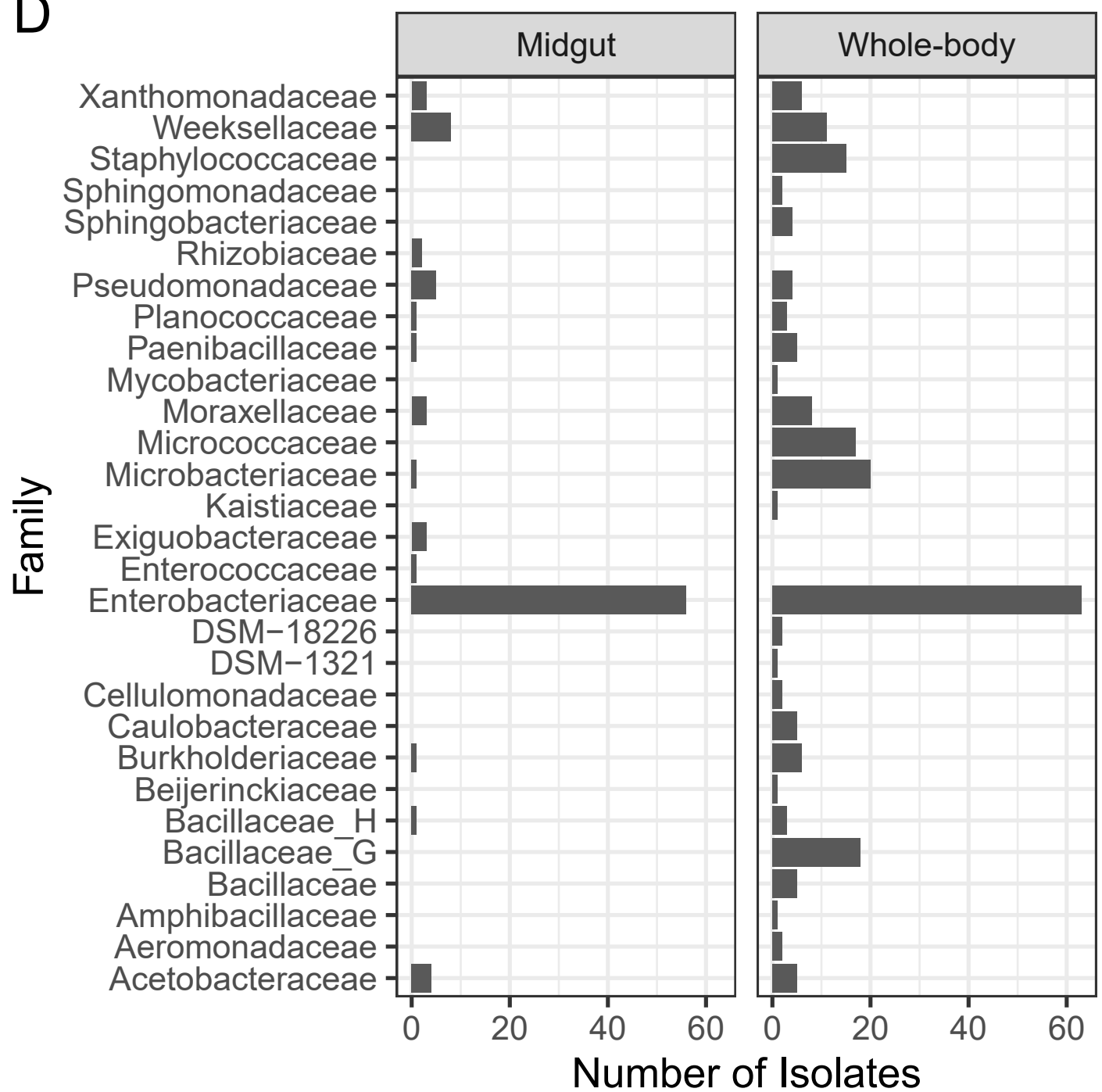

Supplement: S4 Fig — The x-axis of each bar chart shows the number of isolates assigned to a reference genome with a given family assigned taxonomy in the GTDB (y-axis). Charts are faceted by metadata category as follows: (A) female_feeding_status, (B) lab_field_derived, (C) mosquito_sex, and (D) mosquito_tissue. Metadata category names and definitions follow those presented in Table S1. All code and data to recreate this figure can be found at https://github.com/MosAIC-Collection/MosAIC_V1 in folder “12_Metadata_Exploration.” (PDF) [file pbio.3002897.s012.pdf]

A

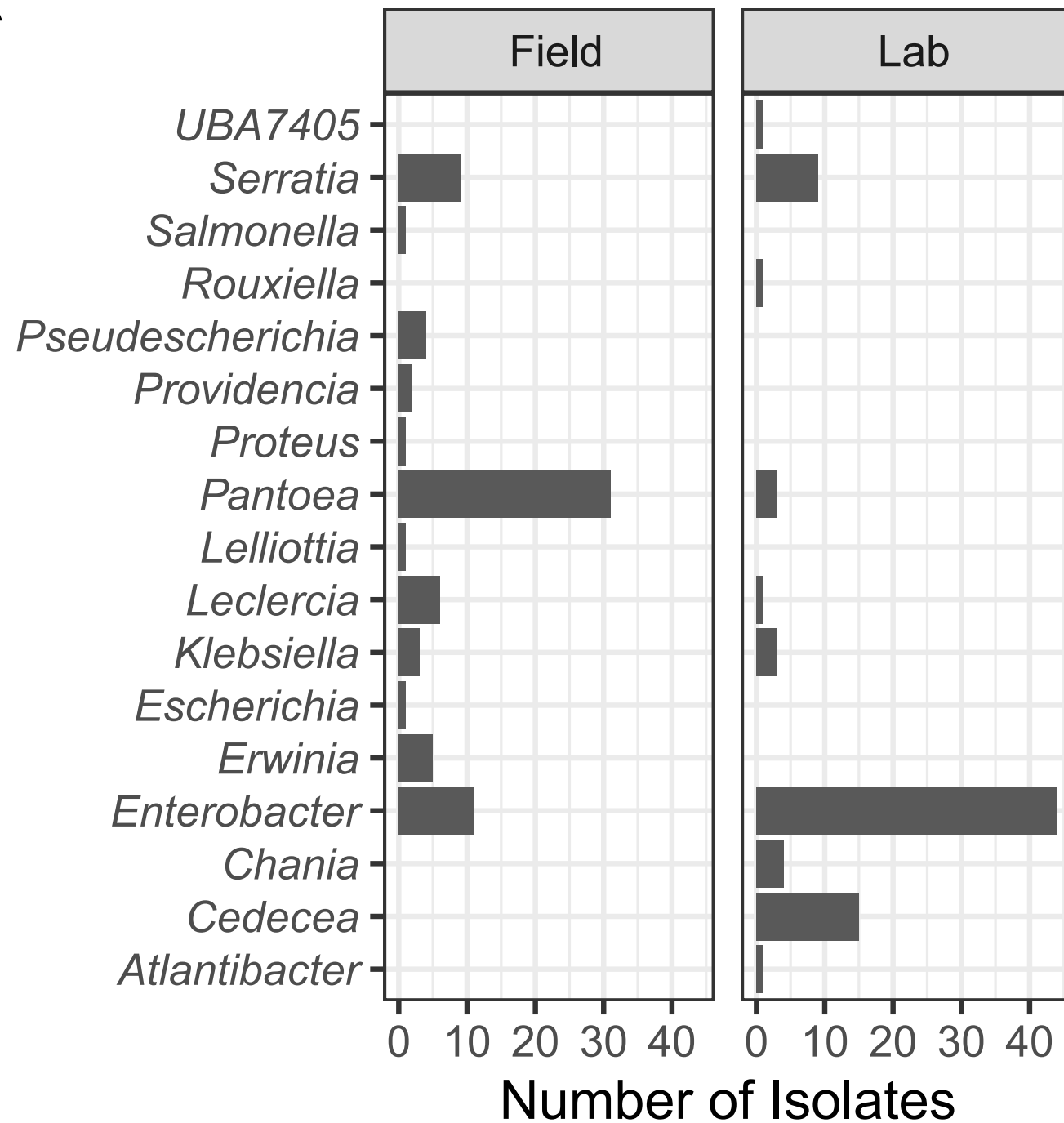

B

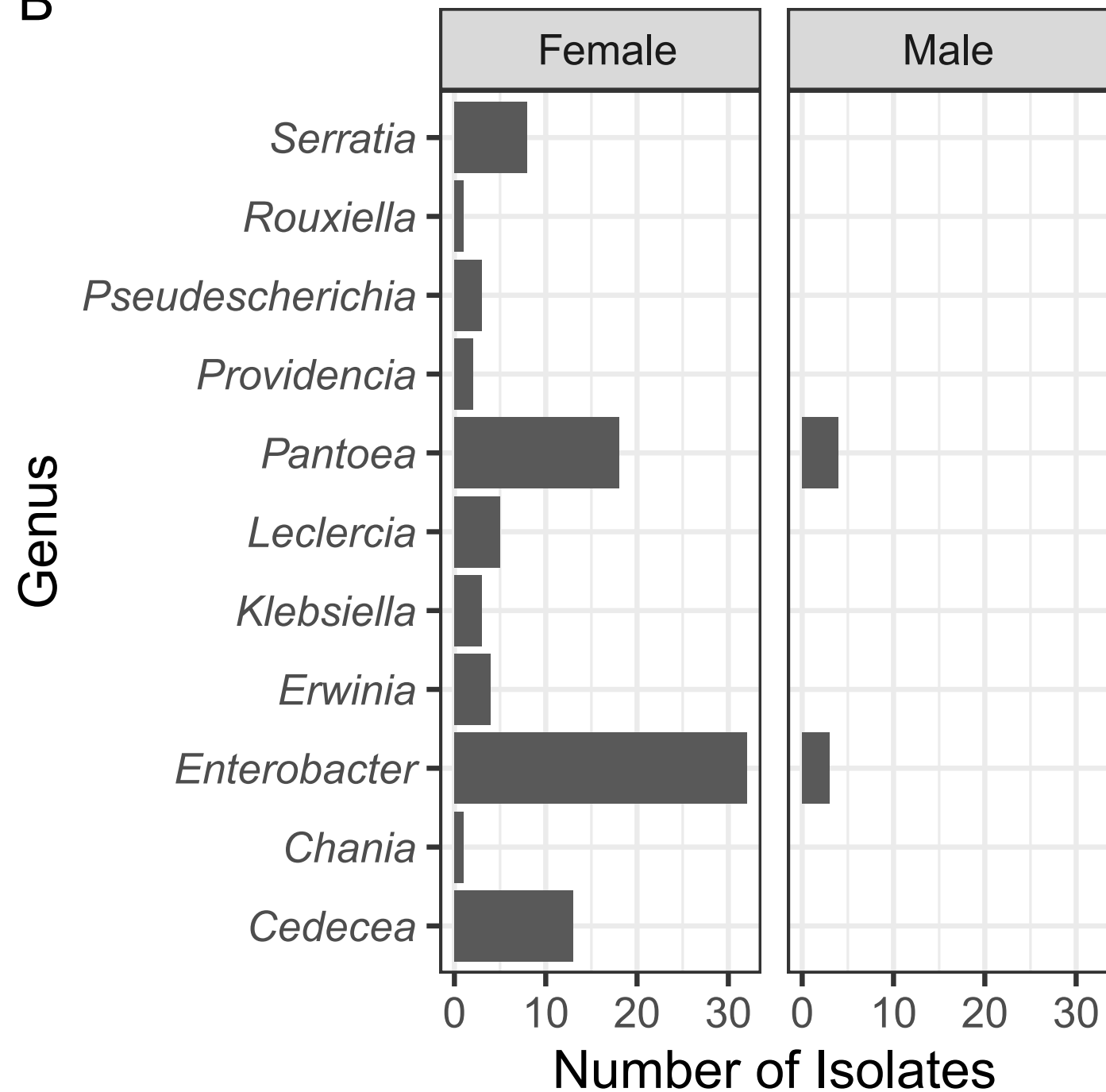

Supplement: S5 Fig — The x-axis of each bar chart shows the number of isolates assigned to a reference genome with a given genus assigned taxonomy in the GTDB (y-axis). Charts are faceted by metadata category as follows: (A) lab_field_derived, and (B) mosquito_sex. Metadata category names and definitions follow those presented in S1 Table. Only isolates assigned to genera within the Enterobacteriaceae were included in the analysis. All code and data to recreate this figure can be found at https://github.com/MosAIC-Collection/MosAIC_V1 in folder “12_Metadata_Exploration.” (PDF) [file pbio.3002897.s013.pdf]

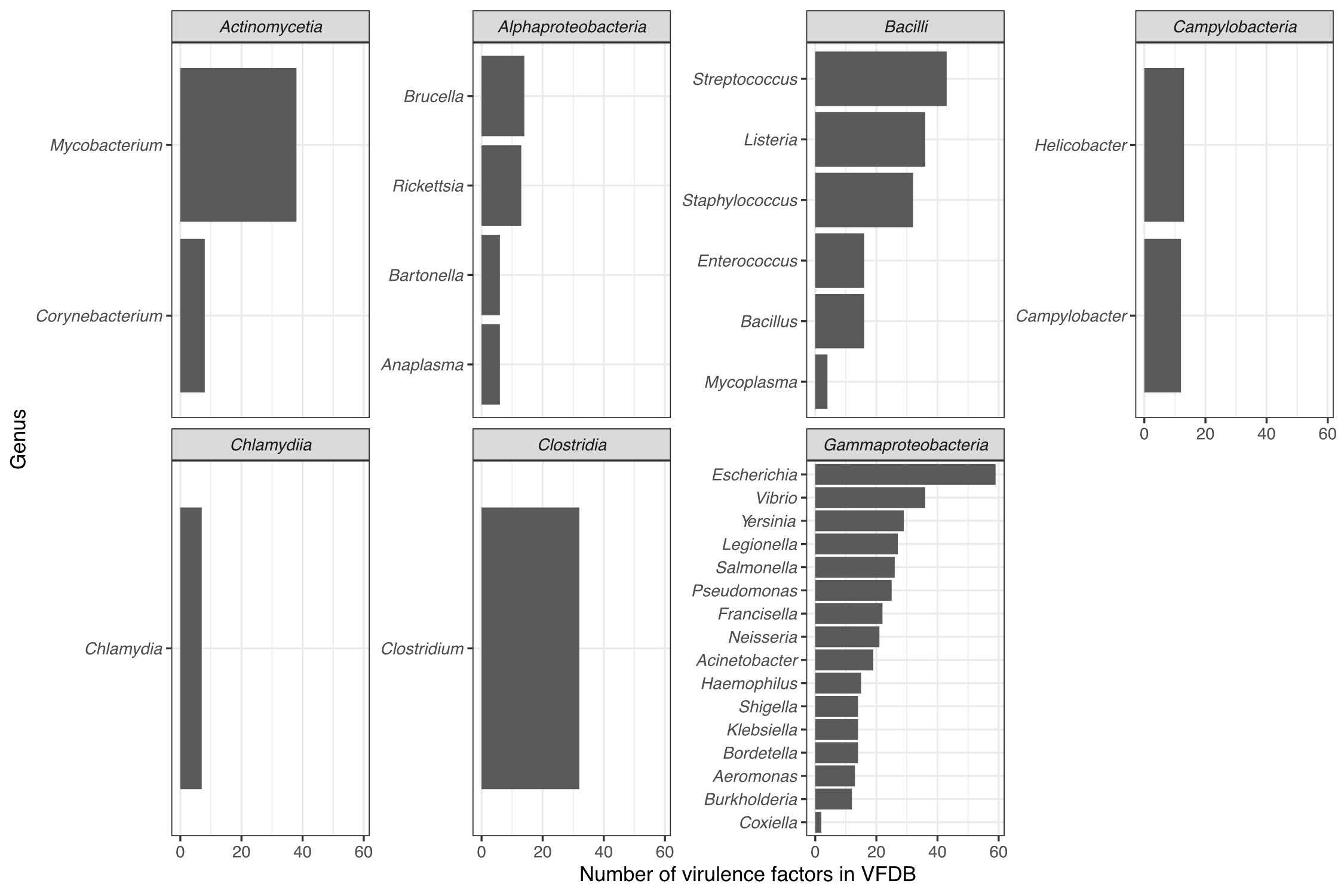

Supplement: S6 Fig — Plots demonstrate the bias in database composition, which shows a strong skew towards members of the Gammaproteobacteria. Plots are faceted by bacterial class, with the x-axis of each chart showing the number of isolates assigned to a given genus on the y-axis in which at least 1 virulence factor gene was identified. All code and data to recreate this figure can be found at https://github.com/MosAIC-Collection/MosAIC_V1 in folder “05_Virulence_Factor_Analysis.” (TIFF) [file pbio.3002897.s014.tiff]

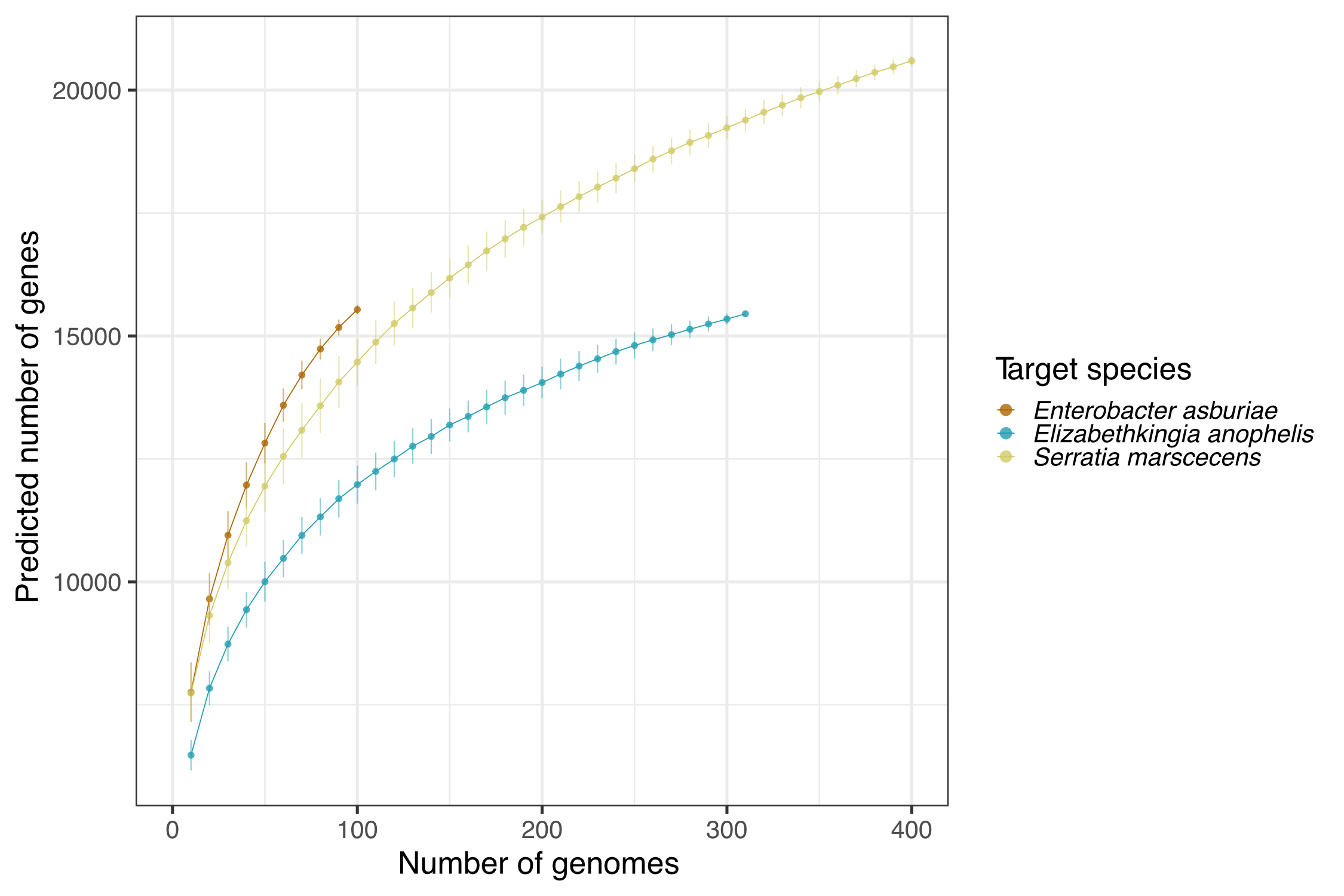

Supplement: S10 Fig — Pangenome gene accumulation curve for En. asburiae, El. anophelis, and S. marcescens isolates from MosAIC. All code and data to recreate this figure can be found at https://github.com/MosAIC-Collection/MosAIC_V1 in folder “08_GeneAccumulationCurve.” (TIFF) [file pbio.3002897.s018.tiff]

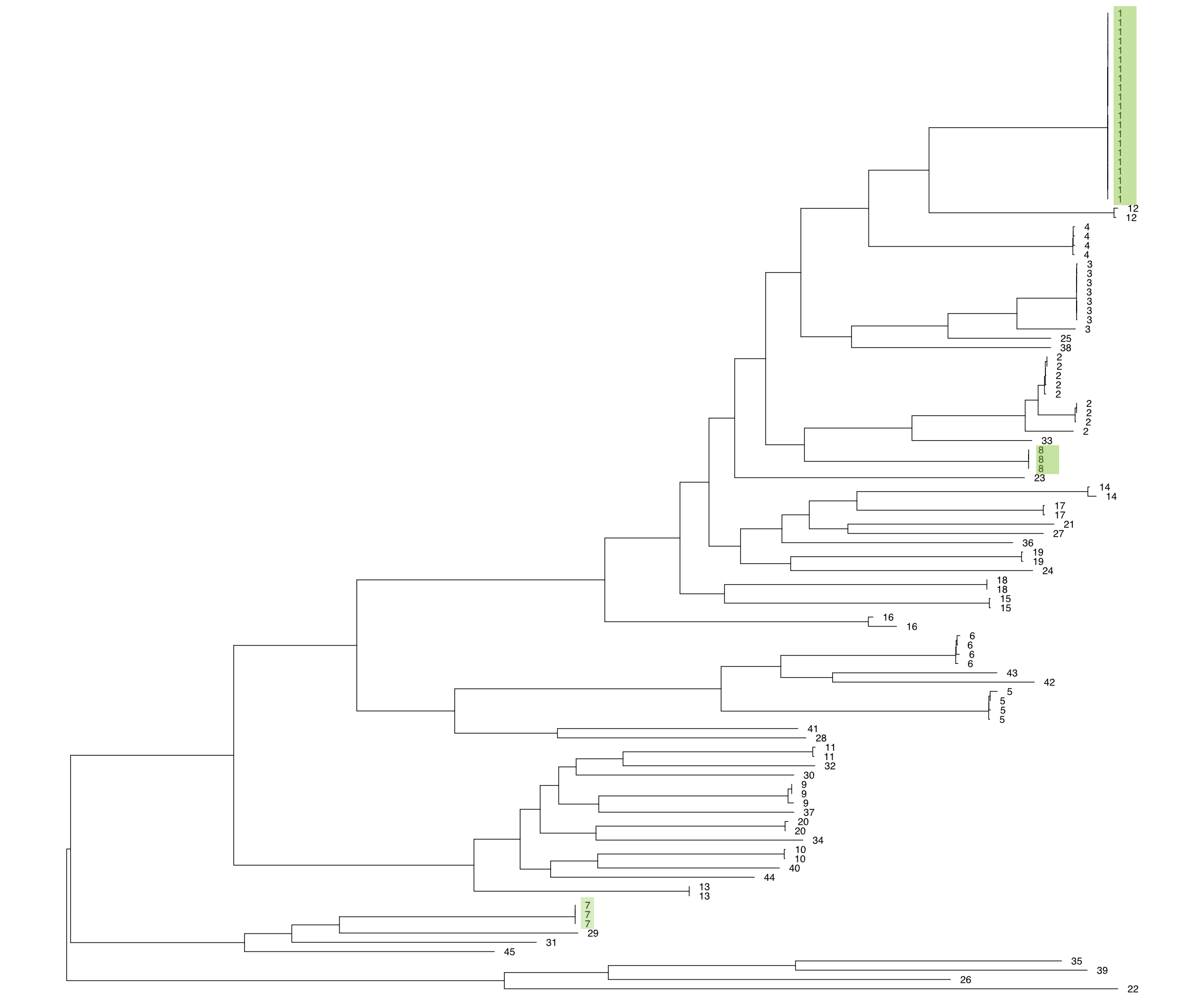

Supplement: S11 Fig — Tips denote PopPUNK cluster. Green highlight denotes mosquito-associated lineages containing MosAIC isolates. All code and data to recreate this figure can be found at https://github.com/MosAIC-Collection/MosAIC_V1 in folder “10_VisPopPUNKClusters.” (TIFF) [file pbio.3002897.s019.tiff]

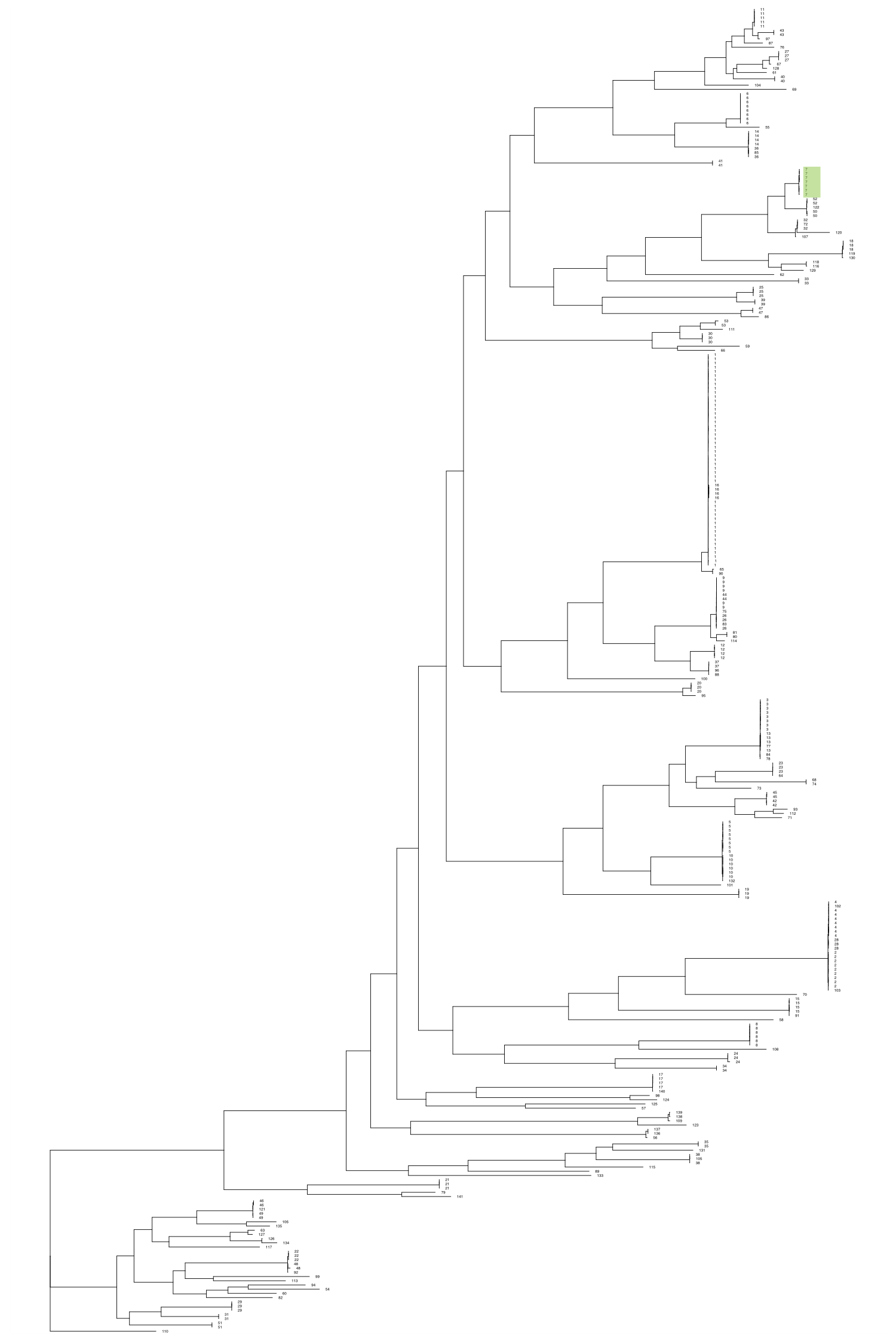

Supplement: S13 Fig — Tips denote PopPUNK cluster. Green highlight denotes mosquito-associated lineages containing MosAIC isolates. All code and data to recreate this figure can be found at https://github.com/MosAIC-Collection/MosAIC_V1 in folder “10_VisPopPUNKClusters.” (TIFF) [file pbio.3002897.s021.tiff]

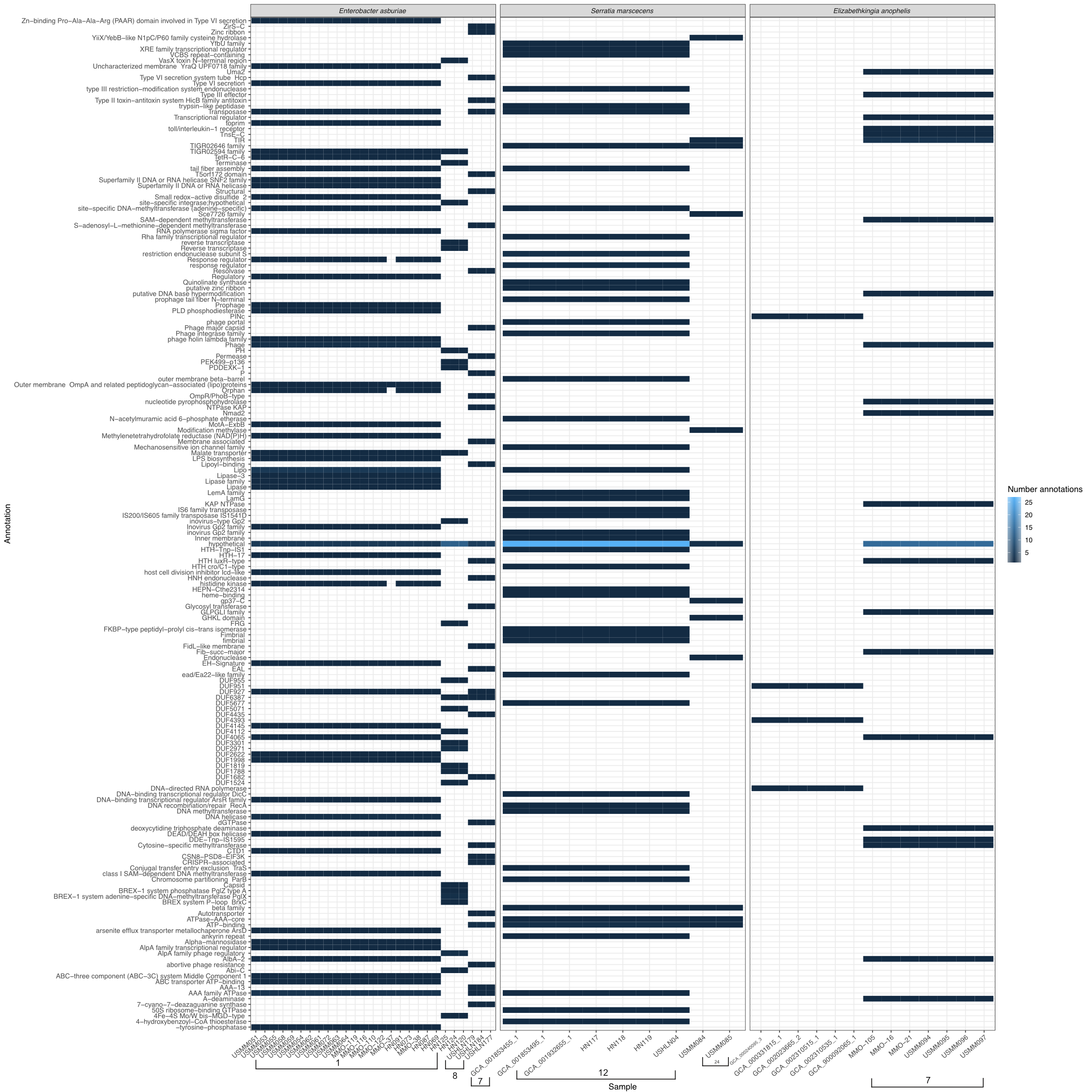

Supplement: S14 Fig — Panels summarise annotations for one of 3 focal species (Elizabethkingia anophelis, left; Serratia marcescens, centre; or Enterobacter asburiae, right), with the x-axis of each panel denoting the internal identifier of individual isolates assigned to each species as presented in S1 Table. Tiles denote the number of identified annotations corresponding to a given functional category on the y-axis, following a gradient from dark blue (few) to light blue (many). White tiles denote categories for which zero annotations were identified in each isolate. All code and data to recreate this figure can be found at https://github.com/MosAIC-Collection/MosAIC_V1 in folder “11_LineageCoreGeneAnalysis.” (TIFF) [file pbio.3002897.s022.tiff]

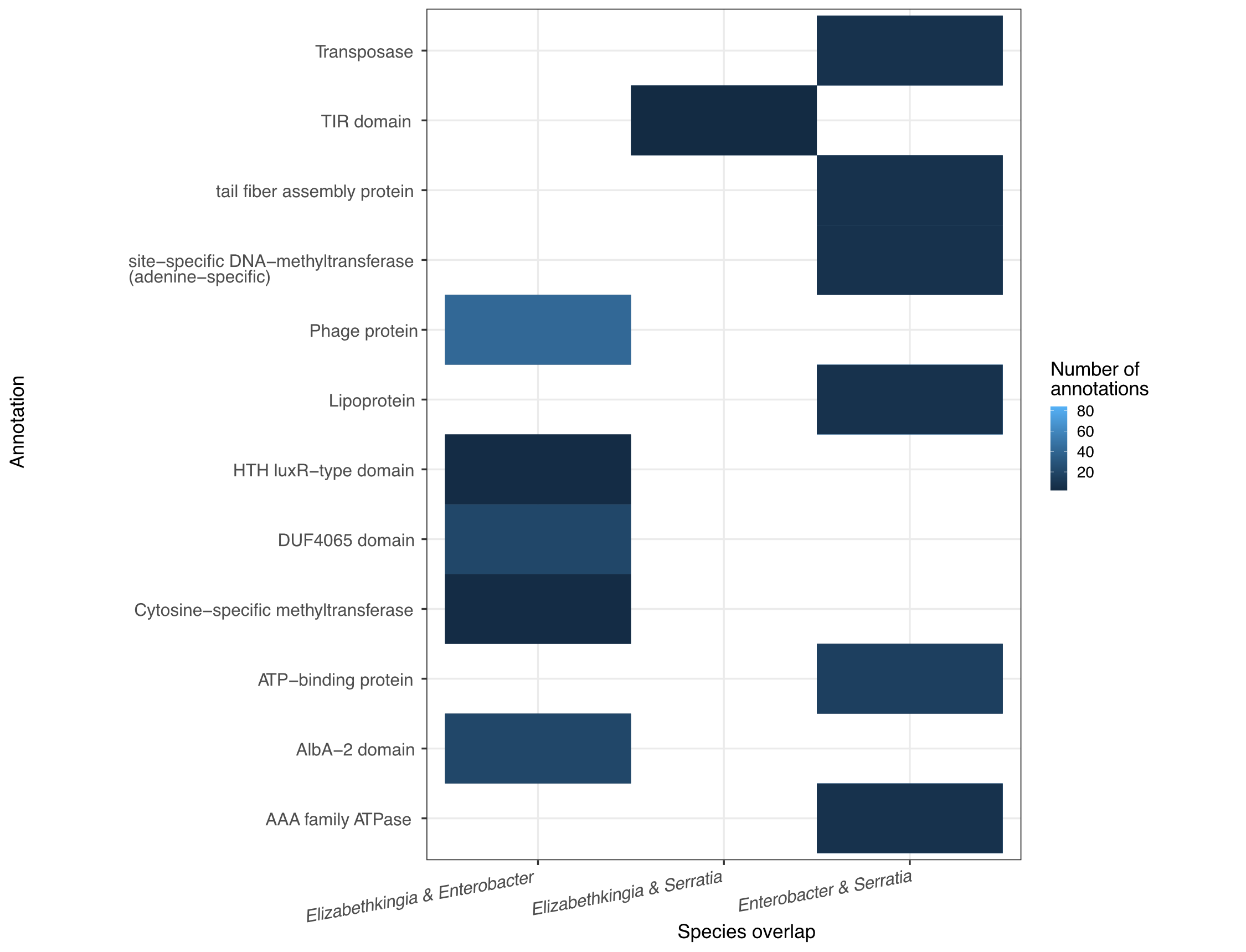

Supplement: S15 Fig — Tiles denote the number of identified annotations corresponding to a given functional category on the y-axis that were shared between a given species pair on the x-axis, following a gradient from dark blue (few) to light blue (many). White tiles denote categories for which zero shared annotations were identified in each species pair. All code and data to recreate this figure can be found at https://github.com/MosAIC-Collection/MosAIC_V1 in folder “11_LineageCoreGeneAnalysis.” (TIFF) [file pbio.3002897.s023.tiff]

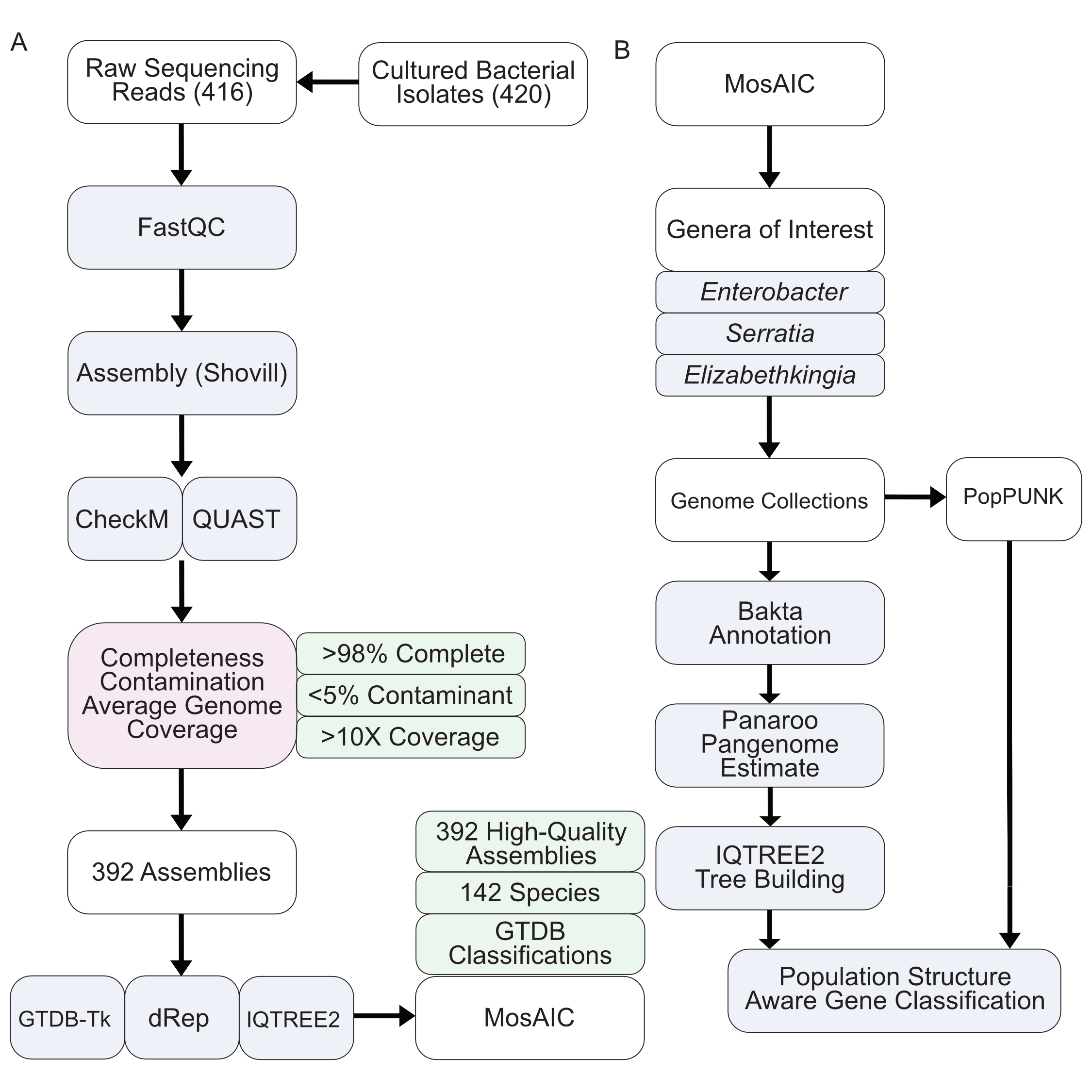

Supplement: S16 Fig — Flowchart describing (A) the assembly of MosAIC genomes and (B) population genomic analyses. (TIFF) [file pbio.3002897.s024.tiff]
