# Supplementary material for: MosAIC: An annotated collection of mosquito-associated bacteria with high-quality genome assemblies
Source: PLoS Biol. 2024 Nov 15;22(11):e3002897. doi: 10.1371/journal.pbio.3002897 (PMC11633956; doi:10.1371/journal.pbio.3002897)
Supplement: Attachment — Submitted filename: MosAIC_reviewers_comments_response_FINAL2.docx [file pbio.3002897.s025.docx]

**PBIOLOGY-D-23-02802R1**

**Response to Reviewers**

Below we write out verbatim the points raised by the reviewers. For each reviewer, we first write out the point they raised (black font) and then follow with our responses (blue font). Note: Provided line numbers reference the manuscript file *with tracked changes* in our resubmission.

We thank the reviewers for their constructive feedback and hope they agree that we have addressed all their questions and concerns in our resubmission.

--

Reviewer #1: Description of Study:

This manuscript describes a collection of bacterial isolates obtained from field and laboratory environments containing mosquitoes. This MosAIC collection contains isolates from several species of mosquitoes and from different geographic localities. In total it has 392 isolates cryopreserved in -80C glycerol stocks with plans to collect more and to diversify in collecting fungi and microbial eukaryotes from mosquito habitats. Additionally, bacterial isolate genomes were individually sequenced with Illumina technology and the manuscript describes the taxonomic diversity, genomic completeness, genetic content focused on virulence factors & pangenome content, and the phylogenetic placement of mosquito-associated isolates among closely related genomes (publicly available) for 3 highly-sampled genera.

               Overall, this is a well-written paper that describes a large collection of bacteria from mosquito-associated environments/guts. This initiative set out to collect isolates in a non-systematic way and therefore could not compare isolates or sampling locations in a statistical framework to draw more information about the associations of different microbial species/genera. This is a resource that has the potential to be used by numerous research groups; however, I feel that before this manuscript can be published there are several things that need to be done. I detail minor and major issues below.

Major Issues essential for publication:

This manuscript is presented as a Resource for the scientific field of host-microbe interactions (specifically mosquito researchers); this is both the physical collection of isolates and the genomic assemblies. This is specifically stated in the Abstract (Lines 56-58). Furthermore, as per the "Data Availability" Section "simply stating 'data available on request from the author' is not acceptable". In reading this manuscript the authors state that the isolates are not yet available in a culture collection (Lines 552-554), and that genome assemblies are not available - only raw data (Lines 653-654). Before publication I believe that 1) the isolates must be deposited and accessioned into a culture collection so that scientists can make requests, and 2) genome sequences for each isolate must be accessioned into GenBank or a similar database to make them public. These steps are important for MosAIC to become a resource that is utilized by the broader field and not to be exclusive to the authors of this manuscript. Without these resources (strains and genomes) being made publicly available, this work does not meet the PLoS Biology threshold for "Data Availability".

               I would note that Table S1 lists the genome assembly accessions (Column AW) as "TBC" (I assume an abbreviation of [To be completed]); and that isolates are in the process of submission to a culture collection (Lines: 552-554).This suggests that the authors plan to submit the genomes and isolates - I ask that these tasks are completed before publication.

Citations from above:

Lines: 56-58 of the Abstract: "we generated a bacterial Mosquito-Associated Isolate Collection (MosAIC) consisting of 392 bacterial isolates with extensive metadata and high-quality draft genome assemblies that are publicly available for use by the scientific community."

Lines 552-554: "Physical isolates for all 392 samples that passed our genome quality thresholds are in the process of being deposited for long-term preservation and public access via culture repository and distribution centers based in the U.S. and Europe. Individual isolates are also available upon request."

We apologize for this oversight; all sequencing data are available under BioProject ID (PRJNA1023190), and we have now updated Table S1 to include accessions to the assemblies as well as the raw Illumina reads.

Submission of all isolates to the American Type Culture Collection (ATCC) is underway (see attached letter) and expected to be complete by the end of the year (see timeline below). In the meantime, all isolates are available upon request to Dr. Coon and infrastructure has been implemented to manage incoming requests in Dr. Coon’s lab at UW-Madison (see [<https://kcoonlab.bact.wisc.edu/mosaic/>](https://kcoonlab.bact.wisc.edu/mosaic/)).

Timeline for depositing with ATCC:

| December 2023 – March 2024 | Deposit request submitted (full proposal required for special consideration given collection size); discussions thereafter |
| --- | --- |
| March – April 2024 | Request formally approved by ATCC leadership; negotiations with UW-Madison |
| April – May 2024 | Replication of in-house collection to ensure long-term preservation at UW-Madison; infrastructure development to manage future requests / contributions |
| May 2024 – December 2024 | Preparation and shipment of isolates to ATCC* |
| *Note: Twenty-five glycerol stocks OF EACH ISOLATE must be provided to ATCC (= 9,800 vials total). Upon arrival at ATCC, stocks provided for a given isolate will be tested for quality assurance prior to being formally catalogued and available for distribution to external researchers. This process will be conducted iteratively for batches of 10 isolates at a time, with the first batch of isolates expected to be publicly available via ATCC in August 2024. | |

Minor Issues:

Several isolates in MosAIC are very closely related (often monophyletic) even as they were collected from different laboratories in geographically different locations. This raises the question of "where did these isolates come from?". The manuscript comments on this a bit, but one aspect that wasn't addressed is the relationship of mosquito colonies among labs - are some of them passed mentor-to-mentee, or between collaborators? This provenance is likely a very important aspect of collecting truly independent samples of mosquito-associated microbial communities from laboratory colonies. Related to this topic is the question "do labs use the same diet for feeding mosquitoes?". The dietary products introduced into rearing water is an obvious source of microbial communities - do the labs use the same diets/providers?

               I am not sure if this information is available, but I believe that addressing these issues in the manuscript would be useful for the field of mosquito-microbe interactions. This could be information in table format (diets used by labs) or in a new figure of mosquito colony origins and links between lab colonies. At a minimum, these topics should be mentioned in the manuscript text.

We thank the reviewer for raising this important point. While information regarding the exact provenance of the different laboratory-reared mosquito strains represented across the collection is not available at this time, we now provide coarse information regarding the larval rearing diets currently in use by contributing research groups (see revised Table S1) and address this topic directly, as suggested by the reviewer, in our revised Discussion (see Lines 459-461).

While the genomic resources associated with MosAIC represent a considerable amount of data, I am unsure about how much use it will receive in future research. This is because the authors utilized Illumina technology which does not produce complete genome sequences. With current long-read options available, bacterial genomes are easily sequenced to completion (whole chromosome(s) + plasmids). These complete genomes are very important for comparative genomics because researchers can be confident that they have all genes in that genome, and it allows for work on gene order (synteny) which is important for HGT analysis. Examining Table S1 (column AC) I found that none of the MosAIC genomes are in a single contig (min = 6; max = 1382; average = 114 contigs) and that approximately half of the isolates are in >80 contigs.

We respectively disagree with the reviewer and believe that these data will be essential in facilitating the functional prediction of microbial communities (and quantification of potential microbial functional redundancy) in different mosquito species/life stages, and within their associated habitats.

Indeed, genome sequencing using short-read data is common practice and used widely across the microbial genomics community for larger studies, and there is no evidence reported anywhere that any genes are missed. Moreover, short-read search algorithms like ariba or srst2 (DOI:[10.1099/mgen.0.000131](https://doi.org/10.1099/mgen.0.000131), DOI:[10.1186/s13073-014-0090-6](https://doi.org/10.1186/s13073-014-0090-6)) are highly sensitive and allow read-based detection of genes that can be challenging in assemblies. While complete genomes, using long-read (for better assemblies) and short reads (for accuracy) for each organism would be the desired situation for all research, that is not common, not feasible for this study, and not required to provide the population genome analysis we present. Short-read assemblies of ~100 contigs is standard in microbial genome population studies, as can be seen across the diversity of large-scale genome studies published in various journals, and draft genomes are well established to be more than sufficient for gene prediction and giving insights of functional potential by predicting metabolic and biosynthetic capacities (*e.g.*, see DOI:[10.3389/fbinf.2022.918853](https://doi.org/10.3389/fbinf.2022.918853), DOI:[10.1128/aem.01614-18](https://doi.org/10.1128/aem.01614-18), DOI:[10.1128/msystems.00223-22](https://doi.org/10.1128/msystems.00223-22)).

Reviewer #2: In this manuscript, Foo et al introduce a library of bacterial isolates originating from mosquitoes and their environment, called MosAIC. They sequenced the whole genomes of their 392 isolates, spanning 142 bacterial species. These bacteria belong to classes and families known to be highly represented in mosquitoes. A large proportion of the isolates were isolated from laboratory samples, and some also derived from field samples.

This manuscript is prepared with care, hence I don't have any comments on the introduction, discussion and methods. As it contains a lot of information, I hope that my following comments will help the authors improve the readability in the results.

Figure 2A would benefit from a clearer separation between bacterial classes on the chart. "Points at the tip of bars": do the authors refer to the stars? If so, do they correspond to Figure 2F? In this case, the authors may clarify (for instance, using numbers instead of the stars) Figure 2B: can the authors increase the size of the dots?

Figure 2D: what is N50?

We thank the reviewer for these suggestions and have added numbers instead of stars to Figure 2A and 2F to denote highly representative species. The size of each dot has also been increased and we have added a definition of N50 to the figure legend.

In the text referring to Fig S4A, the authors could more clearly indicate that examples are discussed (several other families are represented in only non-blood fed or only blood fed samples). If I am not mistaken, figure items S4C and S4D are not described in the text.

The paragraph now begins by acknowledging the limitations of the dataset being discussed (see Lines 213-217). Specific reference to Figure S4C,D is also now made (see Lines 230-232), and the language used to describe families exclusive to non-blood-fed vs. blood-fed mosquitoes makes it more explicitly clear that the examples we provide do not represent an exhaustive list of all taxa associated with a particular condition (see Lines 214-222).

Line 253: I don't think that the beginning of the sentence matches well the end. The authors may mean "between bacterial families", or "within bacterial classes"...

The text has been updated to read: “*The distribution of virulence factor categories also varied between bacterial classes*” (see Lines 269-270).

Line 260: Not sure "Fig S6" corresponds to that sentence. Can the authors clarify?

Fig S6 is the correct figure; however, to clarify, we have added the following sentence to the figure legend: “*Plots demonstrate the bias in database composition, which shows a strong skew towards members of the Gammaproteobacteria*.”

Line 274: not Fig4B => Fig 4D, E, or F?

We thank the reviewer for highlighting this typo; the appropriate panel (Figure 4D) is now referenced in our revised manuscript (see Lines 295-296).

Figure 4 is not easy to get at first sight. Even though I acknowledge that this is due to a large amount of provided information, I would suggest:

- to make a visual link between the coloured bows in A and in B / C so that the subsets appear more obviously (and the same in D => E/F and in G => H)

- to use "F" and "L" instead of the circles and stars for field and lab, and 1-9 for the lab references. Otherwise the reader who wants to know from which lab comes an isolate needs to go from the shape to the number, and from the number to the lab name, that's complicated. One step could be easily removed.

- clearly separate Mosquito species 3 from Host and collection in the legends, and clarify what is in A,D, G and what is in the other figure items.

We thank the reviewer for pointing this out and agree with many of their suggestions. In response, we have revised Figure 4 in the following ways:

1. *Paenibacillaceae & Caulobacteraceae* have been recolored for clearer separation;
2. F and L have been added to the outer ring of each phylogeny to denote field (F) and lab (L) derived isolates and each lab is now denoted with a number; and
3. We have also now grouped Collection and Host, and Isolation Source 1, Lab Source 2 and Mosquito species 3 into separate boxes.

Line 331: the numbers of genes don't correspond to those mentioned in Fig A-C. Please clarify in the text and/or figure.

We have now clarified this by adding the following to the Figure 5 legend: “*Numbers of genes given on the x-axis refer to the total number of genes within each pangenome (core + accessory genes*.”

Line 356: two or three in En. Asburiae? Only two appear in fig S11

We thank the reviewer for identifying this error; three is the correct number and we have revised Figure S11 accordingly.

Fig S14: it would be clearer to keep the same order in the three species as in the rest of the manuscript (Enterobacter => Serratia => Elizabethkingia). And wouldn't it make sense to sub classify the isolates of each species into their clusters?

We have reordered the facets of each species for consistency, as suggested (*Enterobacter asburiae => Serratia marcescens => Elizabethkingia anophelis*). Similarly, we have changed the ordering of each sample on the X axis to denote their PopPUNK predicted cluster.

--

Reviewer #3: Dear Authors and Editorial Staff at PLoS Biology,

This is a potentially outstanding contribution to microbiome science. I applaud the selfless and progressive efforts of these scientists, colleagues who share an interest in the mosquito microbiome, to create an open-science resource that will be extremely valuable to our larger community. Overall, I am highly supportive of their efforts and I would like to see this work published quickly. Moreover, I am excited to contribute to and utilize this emerging resource when it is fully-launched.

I do have some critical comments that I would like to see addressed before this is ultimately accepted for publication. Many of my most critical comments appear to be well-understood by the authors, particularly the limitation of the dataset imposed by the sampling design. My major suggestion is that the authors pivot in the discussion to discuss key features of this proposed resource that must be developed, rather than discussing weakly-substantiated scientific observations that at this stage remain relatively dubious, particularly those that generalize features of mosquito symbiont genomes and function.

Nevertheless, I encourage the editor to work with these authors on this important manuscript. I am optimistic that a resubmission after some thought on my comments below could be published at PLoS Biology.

Overall comments.

This dataset is highly flawed, specifically in its ability to generalize features of the bacterial genome shared among mosquito symbionts. Typical of haphazard collection designs, the dataset is unbalanced with respect to host and environmental substrates, host taxa (mosquito or non-mosquito), mosquito species, field or laboratory populations, host life stage, geography, and host sex. A review of the supplemental files reveals that accounting for this imbalance statistically would be extremely difficult and underpowered. Though the authors share my trepidation in the interpretation of this dataset (e.g. they rightfully resist significance testing and are very clear about the datasets limitations on identifying general genomic features of mosquito-associated symbionts), they utilize a substantial portion of the results and discussion to highlight these comparative patterns found with this dataset.  At best, these remain untested hypotheses based on observations. While these observations form the foundation for much of biological inquiring, it is too early in that process for publication based solely on these merits alone.

With that being said, this is a tremendous resource and a superb example of collaborative science to solve a problem limiting progress in an important and popular field. It is very worthy of publication on these merits alone, and I am thoroughly impressed with the efforts of these scientists to coordinate a resource like this. As a "Methods and Resources" contribution to PLOS Biology, I would like to see the discussion about the growth of this resource emphasized. The authors are the best positioned to guide the community on fundamental and open-ended questions on the functionality of MosAIC, how it can be used, and how it can be improved. Specifically, I pose the following questions and ask the authors to clarify these in a resubmission.

1. Ideally, what is the detailed plan to make these samples available to other researchers? How will these isolates be maintained? How will they be distributed? Who will make the decision on this access and distribution?

See response to Reviewer #1 above. In brief, all 392 isolates within the current collection will be deposited in the American Type Culture Collection (ATCC) for long-term maintenance and distribution. Researchers (especially those from low- and middle-income countries) can also request isolates directly from Dr. Coon’s lab at UW-Madison. The in-house collection at UW-Madison currently consists of duplicated stocks of each isolate in each of two freezers maintained in two separate building on separate power supplies; this ensures long-term maintenance of the collection independent of ATCC. More information can be found on the MosAIC website: <https://kcoonlab.bact.wisc.edu/mosaic/>.

2. How are issues with data sovereignty integrated into the expansion plan? For instance, who owns the isolates? If IP is generated, where do the resulting resources go? Are communities from areas where the isolates were sourced integrated into these plans?

See information outlined on our website (<https://kcoonlab.bact.wisc.edu/mosaic/>). We are committed to working against parachute research (research from high-income countries taking isolates / data from low-income countries without adequate involvement of researchers or communities in country) and intend to honor the object and purpose of existing frameworks for equitable access and benefit sharing related to biological resources, including the Nagoya Protocol on Access and Benefit-Sharing.

In addition to continuing to make all isolates and genome assemblies associated with the collection publicly available, we are also committed to ensuring that:

- All MosAIC data continues to be published in open access journals; and
- All MosAIC contributors and associated personnel will continue to be offered authorship on relevant scientific papers related to the collection and its expansion at UW-Madison.

3. What data are needed to expand the scientific or hypothesis-testing value of the dataset? If the authors would see an influx of isolate submission, where would they hope these isolates are coming from? What dimensions of the metadata remain inadequately sampled and limit our ability to use these data to uncover real patterns among in the genomes and functions of mosquito-associated microbial symbionts?

We have expanded the text to include the following section: “*Future targeted sampling will focus on obtaining isolates from underrepresented regions, in particular inviting contributions from researchers in the global south and regions with endemic mosquito-borne disease. We will also target fungi, archaea, and protozoa, which are currently lacking in the collection*” (see Lines 523-526). A detailed outline of the next iteration will be the next work package for members of our team but goes beyond this publication.

In summary, I am highly supportive of this work. In a resubmission, I'd like to see a deemphasis on (but not necessarily a total abandonment of) the observational analysis of the existing dataset, in favor of more discussion on how this resource should be/will be implemented and grown.

Specific comments for consideration.

Line 106- The use of "we" might be reconsidered as the author list of the cited manuscripts overlaps but does not exhaust the authors of this contribution.

This sentence has been amended in our revised manuscript to read: “*…,members of our team recently determined…*” (see Lines 106-107).

Line 140- It would be helpful to add the number of bacteria isolates obtained from hosts generated in a lab-setting versus those sampled from nature in the main text.

We have now added the following sentence: “*Of these isolates, 169 were derived from hosts maintained in a laboratory setting and 126 were derived from field collections*” (see Lines 145-146).

Line 148/Figure 1. While visually interesting, I find this figure difficult to interpret. It would seem like this was created in order to aid the reader that might be interested, for instance, in the number of isolates from  "Ae. albopictus female mosquitoes collected from the lab that were blood-fed". However, this is challenging to determine qualitatively from the figure and impossible to determine quantitatively. It seems a boring table might be more effective here. Consider replacing the figure with a table.

Thank you for this suggestion. We have added a complementary table (now Table S2) to ensure that the content presented in Fig 1 is accessible to all readers.

Line 169- Please define "ANI" as Average Nucleotide Identity here in the main text, as this is the first place it is introduced to the reader.

This has now been added to the text “*Sixty-two genomes shared >99% average nucleotide identity (ANI) with GTDB reference genomes,…*” (see Lines 174-175).

Line 206- Given the sampling regime, I wonder if this statement has enough support to actually state. I fear it could have a misleading implication.

We have now rephrased this as follows: “*As this initial collection was derived from existing samples and not under any specific sampling framework, it is not possible to draw conclusions regarding bacterial taxa that may be associated exclusively with particular sample types or conditions. However, we do note some interesting observations from the collection presented to date, including the observation that some taxa, including members of the Acetobacteraceae, Bacillaceae_G, and Caulobacteraceae, among others, were only isolated from non-blood-fed female and male adult mosquitoes, while members of other taxa such as Sphingobacteriaceae and DSM-1321 were only isolated from blood-fed females*” (see Lines 213-222).

Line 246. Can you clarify "uniquely present genes" (i.e., unique to what? Class? Family? etc)?

This has now been clarified as “…*genes that appeared in only one sample*” (see Lines 264-265).

Line 271. Which environmental niches are represented among these isolates?

We have now added additional detail to this sentence and have also referenced the supplementary table containing the complete metadata for all publicly available data included in our analyses (see Lines 290-294).

Line 301-318. When one states "laboratory", is that meant to state the researchers that isolated the bacteria regardless of its source in a host from nature or a lab colony, or is it derived from a specific lab colony?

Here we refer to the researchers with a laboratory group, as opposed to a specific laboratory colony. To clarify, we have changed the wording as follows: “*...and were isolated by two different, geographically distinct, laboratories (research groups)*” (see Lines 332-333).

Line 406. "The microbiome is reassembled-resulting in altering composition across life stages". The use of "altering" in this sentence is awkward. Consider revision.

This has now been edited as follows: *“...resulting in changes in community composition across life stages*” (see Lines 453-454).

Line 422-429. Might this change with more balanced sampling design? How do we know that these patterns are not the resent of bias sampling or hierarchies/sample dependencies in dataset?

This has now been rephrased as follows: “*Considering the collection to date, we have observed lineages of solely mosquito-associated isolates within populations of three taxa: Serratia, Enterobacter, and El. anophelis. While further, balanced, sampling efforts are needed to draw conclusions, the observation that these mosquito-associated lineages comprised isolates derived from different mosquito species and geographically distinct locations suggests potential adaptation to the mosquito environment”* (see Lines 458-463).

Line 478. If this is to become a community resource, this will be the role of the whole scientific community interested in mosquito symbionts. I would recommend using more of this manuscript (specifically, the discussion) to lay out a specific plan for this. These authors know the limitation of their data better than anyone else. I believe they should set the narrative and call-to-action to shore up the current inadequacies of this dataset.

See response to Major Point #2 above and expanded content in Lines 533-538 of our revised Discussion.

Line 482. I think it's worth mentioning that this is a well-established and reasonably supported hypothesis in "macro-organismal" community ecology. The support is particularly strong in plant communities and aquatic pool communities. Functional traits tend to underlie more predictable patterns of community assembly (i.e. determinism). In general, microbiome science (particularly those with associated with mosquito vectors) should do a better job at integrating and synthesizing this body of theory as we study microbial communities.

We thank the reviewer for this point and have added the following: *“…as has been postulated in other micro-systems and is comparatively well-studied in the field of macro-ecology*” (see Lines 532-533).

Line 508- As worded, it suggests isolates were derived from environments rather than mosquitoes from diverse environments. Consider clarifying.

This has now been edited for clarity as follows: “*isolates were recovered from a diversity of mosquito species and mosquito-associated environments…*” (see Lines 567-568).

Line 509-516. Lack of systematic sampling is evident here.

We thank the reviewer for this observation; see also our expansion of the text regarding the comment (Lines 213-217).

Line 551-554- This should be a feature of the main text. As a resource contribution, the authors should spend more time on the current accessibility of this resource and the vision to expand access to the scientific community.

Our revised Data Availability section now provides details for how to access all MosAIC resources:

“*Physical isolates for all 392 bacterial strains in the collection have been deposited for long-term preservation and public access in the American Type Culture Collection (ATCC; Gaithersburg, MD, USA). Isolates are also available upon request (see* [*https://kcoonlab.bact.wisc.edu/mosaic/*](https://kcoonlab.bact.wisc.edu/mosaic/)*).*

*Raw Illumina reads and assemblies are available in the NCBI Sequence Read Archive (*[*https://www.ncbi.nlm.nih.gov/sra*](https://www.ncbi.nlm.nih.gov/sra)*) under BioProject ID PRJNA1023190 (see accessions in Table S1). Scripts used for analysis and figure generation are available in MosAIC’s GitHub Repository (*[*https://github.com/MosAIC-Collection/MosAIC_V1*](https://github.com/MosAIC-Collection/MosAIC_V1)*). All other relevant data supporting the findings of this study, including isolate-specific metadata, are available within the article and its supplementary information.*” (see Lines 707-716).

As noted above, additional information regarding our vision for the collection’s expansion is now provided in Lines 533-538 of our revised Discussion.
