# Supplementary material for: MosAIC: An annotated collection of mosquito-associated bacteria with high-quality genome assemblies
Source: PLoS Biol. 2024 Nov 15;22(11):e3002897. doi: 10.1371/journal.pbio.3002897 (PMC11633956; doi:10.1371/journal.pbio.3002897)
Supplement: Attachment — Submitted filename: response_to_reviewers_01102024.docx [file pbio.3002897.s026.docx]

**PBIOLOGY-D-23-02802R2**

**Response to Reviewers**

We thank the editor and original and new reviewer for their comments. Please find our responses below (blue font).

--

Based on the reviews, the Academic Editor's assessment of your revision, and feedback from an additional reviewer regarding Reviewer #3’s previous concerns, there are still key points that must be addressed before we can proceed with acceptance. One major issue is the overestimation of the observational analysis of the existing dataset, which was not adequately revised or discussed, as requested by Reviewer #3. As also pointed out from the additional comment, which you can find below, the biological conclusions are not solid, and therefore must be deemphasized and properly discussed in the main text. Furthermore, the Academic Editor has highlighted Reviewer #1’s concern about the potential common origin of isolates from lab colonies, as mosquito colonies are often shared between labs. While we do not expect a full resolution of this issue, it is essential to acknowledge it in the discussion section; although it is not expected to fully address this question, we required that you mention this in the discussion. I would like to emphasize that without these adjusments in the text, we will not be able to move towards publication.

As outlined below, we have taken additional steps in our revised manuscript to further de-emphasize the biological significance of our analyses and to be even more explicit about the biases introduced by our non-systematic approach to sampling.

Our initial revision did include added language in the Discussion to address Reviewer #1’s concern about the potential common origin of isolates from lab colonies, which the reviewer appreciated (see comments below). Nevertheless, we have expanded on these discussion points in our second revision to provide additional context as to why this does not represent a major limitation of our study (see Lines 526-536).

We have also further edited Table S1 to include the geographic origin of all laboratory strains of mosquitoes sampled in the current study and to make clear that the only mosquito strain sampled across different laboratory environments (the *Aedes aegypti* Liverpool strain), while derived from the same parental colony, has since been maintained continuously in isolation within these different laboratories for >30 years. Edits to Figure 4 have additionally been made to more clearly delineate isolates by laboratory source.

Overall, we have taken numerous opportunities to emphasize the need to be conservative in the interpretation of our analyses. This includes content within every section of the main text, e.g.:

- Lines 68-74
- Lines 129-134
- Lines 227-229
- Lines 291-294
- Lines 495-498
- Lines 536-538
- Lines 641-646

(List not exhaustive)

We hope that the changes outlined herein, along with the fact that we have submitted our manuscript as a “Methods & Resources” article—an article type for which PLoS Biology does not require the presentation of significant new biological insights (see <https://journals.plos.org/plosbiology/s/what-we-publish#loc-Research-based-content>)—have made our revised manuscript suitable for publication in the journal.

REVIEWS:

Reviewer #1: The authors have made sufficient revisions on this manuscript. It is well written and provides a major resource that is available to the research community. This data will help researchers create hypotheses about genes/gene clusters found in mosquito-associated bacteria. It will also provide a location for future researchers to submit isolates from diverse locations and mosquito species.

We thank the reviewer for their positive comments.

----------------------------------------------------------------

ADDITIONAL REVIEWER COMMENTS:

My main concern is that most of the paper is the description and comparative analysis of the genomes on the collection. Given the biased sampling and lack of an a priori design to perform these comparisons the conclusion cannot be solid. The authors alert for this bias and do not perform proper statistical analysis of these comparisons but, nonetheless extensively duel on it [figure 5 is very difficult to understand in terms of gene classification categories. I cannot really understand the figure]. These analyses would not grant publication by themselves and have very weak biological significance as they are. Mostly, they are based on lab isolates that are extremely similar. Even if they come from different labs they may have been shared recently between them. Core genome and shared genes analysis of these extremely similar bacteria is not very interesting. The third reviewer exposes this in slightly different words: “…rather than discussing weakly-substantiated scientific observations that at this stage remain relatively dubious, particularly those that generalize features of mosquito symbiont genomes and function.“ The authors addressed only later, specific comments of reviewer 3 on scientific meaning, and with basically cosmetic changes.

We have now further adjusted the text to better address the points raised. In particular, we now: (i) clarify the origin of the relevant data better (i.e., whether mosquito-derived isolates were from lab colonies or field-collected mosquitoes), (ii) provide additional discussion of the potential impact of lab colonies in different labs being derived from the same original colony, and (iii) highlight the biased nature of our collection more prominently in the Discussion.

-This can now be found in (i) Lines 140-141, (ii) Lines 526-533, and (iii) Lines 641-644

We have also deleted and/or edited text throughout the Discussion to further de-emphasize the biological significance of our analyses (see Lines 495-496). See response to Academic Editor above for more information.

Finally, please also address the following editorial concerns:

a) We routinely suggest changes to titles to ensure maximum accessibility for a broad, non-specialist readership, and to ensure they reflect the contents of the paper. In this case, we would suggest a minor edit to the title, as follows. Please ensure you change both the manuscript file and the online submission system, as they need to match for final acceptance:

"MosAIC: an annotated collection of mosquito-associated bacteria with high-quality genome assemblies"

The title of our revised manuscript has been changed as suggested.

b) Please mention in the abstract that the bacterial isolates are publicly available as a resource to the community

Done as suggested (see Lines 59-62).

c) Thank you for providing the scripts to generate the figures. However, please state clearly how to use the scripts and to which figure they are useful. According to our PLOS Data Policy, we require that all data be made available without restriction

Our GitHub repository now includes a README file (Lines 853-854) that outlines how repository code/data can be used to reproduce all figures in the manuscript.

Please make sure that you supply the necessary data and scripts for the following figures:
Figure 2BCDE, 3, S1, S2, S4ABCD, S5AB, S6, S10, S14, S15
NOTE: the numerical data provided should include all replicates AND the way in which the plotted mean and errors were derived (it should not present only the mean/average values).
As well, as the tree files, or the necessary data to build them, for figures 2A, S7, S8, S9. S11, S12, S13

All code/data needed to reproduce each of the referenced figures are available in MosAIC’s GitHub repository (for which we now provide a permanent DOI via Zenodo (<https://zenodo.org/doi/10.5281/zenodo.13861761>) and/or within the manuscript and its supplementary information.

d) Please cite the location of the data clearly in all relevant main and supplementary Figure legends, e.g. “The data underlying this Figure can be found in S1 Data” or “The data underlying this Figure can be found in”

We now include a supplemental file (File S2) that outlines how repository code/data can be used to reproduce all figures in the manuscript (see Lines 843-854 for updated “Data availability” statement).

e) Many thanks for providing the underlying scripts in GitHub. However, because Github depositions can be readily changed or deleted, please make a permanent DOI’d copy (e.g. in Zenodo) and provide this URL in the manuscript and Data Availability Statement. I have asked my colleagues to include this request alongside their own.

Done (see Lines 843-854 for updated “Data availability” statement).

f) Please ensure that your Data Statement in the submission system accurately describes where your data can be found and is in final format, as it will be published as written there.

Done.

g) Per journal policy, if you have generated any custom code during the course of this investigation, please make it available without restrictions upon publication. Please ensure that the code is sufficiently well documented and reusable, and that your Data Statement in the Editorial Manager submission system accurately describes where your code can be found.

Done (see responses above).

Please note that we cannot accept sole deposition of code in GitHub, as this could be changed after publication. However, you can archive this version of your publicly available GitHub code to Zenodo. Once you do this, it will generate a DOI number, which you will need to provide in the Data Accessibility Statement (you are welcome to also provide the GitHub access information).

Done (see responses above).
